# Deep Sequencing of RNA from Ancient Maize Kernels

**DOI:** 10.1371/journal.pone.0050961

**Published:** 2013-01-11

**Authors:** Sarah L. Fordyce, Maria C. Ávila-Arcos, Morten Rasmussen, Enrico Cappellini, J. Alberto Romero-Navarro, Nathan Wales, David E. Alquezar-Planas, Steven Penfield, Terence A. Brown, Jean-Philippe Vielle-Calzada, Rafael Montiel, Tina Jørgensen, Nancy Odegaard, Michael Jacobs, Bernardo Arriaza, Thomas F. G. Higham, Christopher Bronk Ramsey, Eske Willerslev, M. Thomas P. Gilbert

**Affiliations:** 1 Centre for GeoGenetics, Natural History Museum of Denmark, Copenhagen, Denmark; 2 Universidad Nacional Autónoma de México, Cuernavaca, Morelos, Mexico; 3 Department of Anthropology, University of Connecticut, Storrs, Connecticut, United States of America; 4 Centre for Novel Agricultural Products, Department of Biology, University of York, York, United Kingdom; 5 Manchester Interdisciplinary Biocentre, Faculty of Life Sciences, University of Manchester, Manchester, United Kingdom; 6 Laboratorio Nacional de Genómica para la Biodiversidad, CINVESTAV-IPN, Irapuato, Guanajuato, Mexico; 7 Arizona State Museum, University of Arizona, Tucson, Arizona, United States of America; 8 Instituto de Alta Investigación, Departamento de Antropología, Centro de Investigaciones del Hombre en el Desierto, Universidad de Tarapacá, Arica, Chile; 9 Research Laboratory for Archaeology and the History of Art, Oxford, United Kingdom; University College London, United Kingdom

## Abstract

The characterization of biomolecules from ancient samples can shed otherwise unobtainable insights into the past. Despite the fundamental role of transcriptomal change in evolution, the potential of ancient RNA remains unexploited – perhaps due to dogma associated with the fragility of RNA. We hypothesize that seeds offer a plausible refuge for long-term RNA survival, due to the fundamental role of RNA during seed germination. Using RNA-Seq on cDNA synthesized from nucleic acid extracts, we validate this hypothesis through demonstration of partial transcriptomal recovery from two sources of ancient maize kernels. The results suggest that ancient seed transcriptomics may offer a powerful new tool with which to study plant domestication.

## Introduction

Small changes at the DNA level frequently result in profound and unpredictable changes at the RNA level. Therefore, transcriptomics - the genome-wide expression profiling of RNA – plays a fundamental role of describing evolutionary change, at the molecular, cellular, and phenotypic levels [1]. Some of the most well-documented effects of transcriptomic change concern crops such as maize (*Zea mays* spp. *mays*), that have undergone extensive morphological changes over the past 10,000 years as a consequence of domestication [2]–[5]. The domestication process itself is of key interest in archaeological science, and ancient DNA studies of desiccated maize remains have offered important insights such as characterizing the timing of selection for traits [6], and documenting the spread of cultivation [7].

Kernels are one of the most relevant tissues for study in maize breeding and evolution: they contain the nutritional value, and are well-preserved tissues to study as ancient remains [8]. Hence, the characterization of RNA from ancient maize kernels would greatly complement and extend the scope of domestication studies, while unlike the genome, which is generally fixed for a cell line, the transcriptome can vary as a result of external environmental conditions. However, it is unclear whether transcripts appearing in ancient samples would reflect the true composition of transcripts when the plant was alive. Furthermore, the transcriptome reflects the genes, which are being actively expressed at any given time (excluding mutations), allowing the characterization of the functional evolution associated with domestication.

Additionally, the transcriptome could enable the reconstruction of the tempo, and strength, of up/down regulation of protein expression and its relation with phenotypic features, such as morphology, nutritional value, and even pathogen defense. Current analyses in maize leaf trancriptomics [9], for example, have shown that it is possible to exploit the continuous developmental gradient in one tissue to investigate spatial differentiation. Furthermore, modern transcriptomics has also proven relevant for a series of features in model organisms of major functional and evolutionary interest for domestic crops. This includes desiccation tolerance in seeds [10], storage protein transcripts [11], endosperm development and starch filling [12], and even seed dormancy and germination, as discussed by [13].

Despite significant technical advances in the field of ancient genetics, recently cumulating in the publication of several complete ancient genomes [14]–[17], reports of ancient RNA are limited. This may be due to the dogma that RNA, being notoriously labile in the laboratory, has little chance of post mortem survival, given both the ubiquitous presence of RNases, and that the 2′-hydroxyl group of RNA is particularly susceptible to hydrolytic degradation [18]. Despite this, circumstantial evidence suggests long-term survival of RNA in some tissues, which could potentially be exploited in the genetic context. One such example is desiccated seeds, which require survival of diverse RNA transcripts for successful germination [19], and thus are likely to have mechanisms to limit the rate of RNA degradation (e.g. RNA chaperones [20]). That this RNA may persist over long time periods has been attested by both nucleic acid hybridization and mass spectrometric detection of short pieces of ancient RNA derived from radish, cress and maize seeds dating back as far as 3,300 years [21]–[23] (although these studies could not rule out contamination or DNA degradation as the source of the detected uracil), and the germination of a 2,000 year old date palm seed [24].

We hypothesize that these observations, coupled with the power of RNA-Seq [1], could in principle, make ancient seed transcriptomics feasible. To test our hypothesis we have explored, through second-generation sequencing (SGS) and bioinformatic characterization, RNA recovered from desiccated maize kernels from Turkey House Ruin in Navajo County, Arizona (Figure S1), dated to 723±23 ^14^C YBP (Supplementary Methods S1, Figure S2 and Table S1).

## Materials and Methods

### The samples

A total of 6 maize kernels were utilized in this study. The kernels (Figure S1) were excavated as a single batch (batch 935) at the Turkey House Ruin, Arizona, in 1915 and have been stored at room temperature at the Arizona State Museum (ASM) since. The maize was examined and identified as white or yellow flint corn and reported to be so uniform that they could have come from 1 ear or from very similar ears [25]. Table 1 contains details of the kernel and sample names.

**Table 1:** Kernel and sample information and names.

Kernel	Nucleic Acids	Sample Name	Batch	Location	Age	SGS Platform
9351	RNA	FLX1	935	Turkey House Ruin, Arizona	723±23 14C YBP	GS FLX
	DNA	FLX4	935	Turkey House Ruin, Arizona	723±23 14C YBP	GS FLX
9352	RNA	FLX2	935	Turkey House Ruin, Arizona	723±23 14C YBP	GS FLX
	DNA	FLX5	935	Turkey House Ruin, Arizona	723±23 14C YBP	GS FLX
9353	RNA	FLX3	935	Turkey House Ruin, Arizona	723±23 14C YBP	GS FLX
	DNA	FLX6	935	Turkey House Ruin, Arizona	723±23 14C YBP	GS FLX
9354	RNA	935130	935	Turkey House Ruin, Arizona	723±23 14C YBP	HiSeq2000
9355	RNA	935230	935	Turkey House Ruin, Arizona	723±23 14C YBP	HiSeq2000
9356	DNA	AZ Shotgun [25]	935	Turkey House Ruin, Arizona	723±23 14C YBP	GAIIx

A total of 6 kernels were utilized for either RNA or DNA extraction only, or co-extracted for DNA and RNA. After nucleic acids were co-extracted, samples were divided into 2 aliquots, and RNA samples were DNase treated and DNA samples were RNase treated (see Methods and Supplementary Methods S1), and labeled with unique sample names.

### Nucleic acid extraction

All extractions and library builds were carried out in a dedicated ancient DNA laboratory, physically separated from any modern maize and amplified nucleic acids. Both DNA and RNA were examined in this study, so that a comparison between the DNA and RNA content of the kernels could be made. Nucleic acid extraction from the ancient maize kernels was performed using an optimized protocol, involving initial bleaching of the testa to minimize external contaminant carryover, endosperm digestion in a customized buffer (Supplementary Methods S1), co-extraction of DNA and RNA using organic solvents, DNase treatment, and complimentary DNA (cDNA) synthesis.

### Complimentary DNA synthesis

Prior to cDNA synthesis, the RNA extracts were treated using DNase I to eliminate the DNA content, following the manufacturer's protocol (Invitrogen, Carlsbad, CA). First-strand cDNA synthesis was performed using random hexamers and a Superscript III First-Strand cDNA Synthesis Kit (Invitrogen), following the manufacturer's instructions. Second-strand synthesis was performed on the cDNA samples, using the Superscript Double-Stranded cDNA Kit (Invitrogen), following the manufacturer's instructions.

### Testing for the presence of DNA and RNA

A series of PCR amplifications were performed to (i) investigate DNA and RNA survival in the kernels, to (ii) verify the efficiency of the extraction method at extracting both DNA and RNA, to (iii) verify the efficacy of the DNase I treatment at removing DNA from the extracts, and (iv) to verify the efficacy of the cDNA synthesis method at reverse transcribing RNA into cDNA. The successful PCR products were not sequenced, thus should only be viewed as indicative of DNA or RNA survival.

Specifically, three primer sets were designed (see Supplementary Methods S1 for primer sequences) to amplify (i) DNA only, (ii) DNA and RNA, and (iii) maize RNA only. cDNA and DNA samples were amplified with all three primer sets. For cDNA samples to be included for sequencing they could only be amplified with the latter two primer sets, thus ensuring that the majority of the cDNA nucleic acid content was RNA. Likewise, for DNA samples to be included, they could only be amplified with the first primer set. Included samples were then converted into libraries for deep sequencing on two different SGS platforms.

### Roche GS FLX sequencing

Firstly, 6 extracts (3 cDNA and 3 DNA, from the same 3 kernels – Table 1) were sequenced on the Roche Genome Sequencer (GS) FLX (Roche, Basel, Switzerland). This platform is capable of sequencing reads of up to ∼500 bp (up to ∼1 kb using FLX+ chemistry), allowing us to assess the fragment length distribution of our ancient maize kernel RNA and DNA (see Table 1).

Double stranded cDNA from the three kernels (Table 1), samples FLX1, FLX2 and FLX3, was constructed into libraries and sequenced on one lane of a GS FLX PicoTiterPlate (PTP) each, following the manufacturer's Multiple Identifier (MID) Library Preparation Protocol (excluding fragmentation and short fragment removal) using LR70 chemistry.

GS FLX libraries were also constructed based on the DNA extracted from the same three maize kernels, and samples were labeled FLX4, FLX5 and FLX6 (Table 1). Prior to library construction, the DNA containing elute was treated with RNase A (Invitrogen) following the manufacturer's protocol, and were then sequenced on the GS FLX in the same manner as the cDNA.

### Illumina HiSeq 2000 sequencing

Secondly, two cDNA samples were sequenced on two lanes of the Illumina HiSeq2000 platform (Illumina, San Diego, CA, USA). Unlike the GS FLX, the conventional read length limit is ∼100 bp. However, the advantage of this platform is that it is able to generate up to 200 Gb per run, allowing us to fully explore the endogenous nucleic acid content of our ancient maize kernels. Double stranded cDNA, from 2 kernels (935130 and 935230), was converted into Illumina HiSeq2000 libraries using the NEBNext Quick DNA Sample Prep Master Mix Set 2 (NEB, Ipswich, MA, USA), following the manufacturer's protocol (excluding fragmentation and small fragment removal steps), and indexed using Multiplex Adapters (Mutliplexing Sample Preparation Oligonucleotide Kit, Illumina). HiSeq2000 sequencing was performed following the manufacturer's protocol for single-read, 100 bp settings.

## Results

### GS FLX sequencing

**Figure 1:**
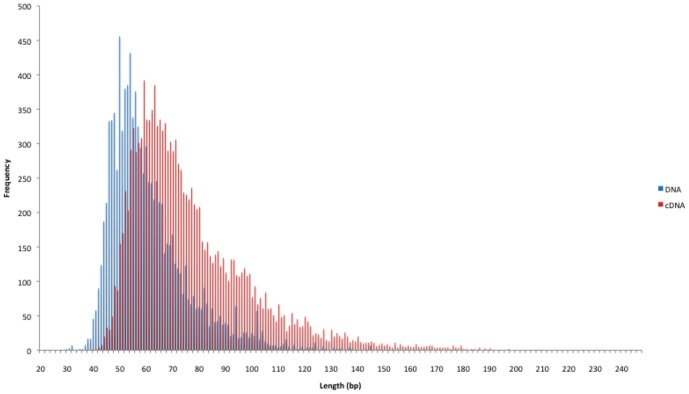
Histogram showing fragment length distribution of maize GS FLX sequence reads from 3 Arizonan DNA (FLX4-6) samples (blue) in comparison to 3 Arizonan cDNA (FLX1-3) samples (red).

Three cDNA and 3 DNA samples were sequenced on the Roche GS FLX, producing a total of 22,338 sequences from all the cDNA libraries (FLX1-3) and a total of 31,010 sequences from all the DNA libraries (FLX4-6). The sequence lengths obtained ranged from 40–247 bp (Fig. 1) for cDNA, and 22–186 bp for DNA, although these lengths cannot be taken at face value due to (i) automatic filtering out of short reads by the GS FLX sequencer software, and (ii) the longest reads being limited in length by the read capacity of the GS FLX sequencer under LR70 chemistry.

### HiSeq 2000 sequencing

Two Arizona kernels (935130 and 935230) were constructed into cDNA libraries and deep sequenced on one lane each using the HiSeq2000. A total of ∼260 million reads were generated from the 2 samples. DNA was sequenced in a previously published study [26], using the Illumina Genome Analyzer (GA) IIx.

### Data analysis

#### Endogenous nucleic acid content analysis

In order to explore the endogenous nucleic acids content, GS FLX reads from all cDNA and DNA samples were converted to fastq format and mapped to the B73 maize genome [27], using a combination of BWA (version 0.5.9-r16) [28] and BLAT [29] (Table S2). Potential PCR duplicates were removed using Samtools' rmdup and ambiguous hits were removed by setting a mapping quality filter of 25 and by controlling for XT and XA tags. Even though the endogenous nucleic acid content was high in both cDNA and DNA libraries (48–92% and 42–68%, respectively), the total yield of non-clonal, uniquely mapped reads was low and did not allow a reliable quantification of gene overlap (Table S2). All bam files were manipulated using Samtools as well as Perl and R scripts.

HiSeq 2000 reads were mapped and processed in the same way as GS FLX reads with the exception that, prior to mapping, reads were manipulated with an in-house script (available on request) to remove sequencing adapters and low-quality stretches in the 3′ end of reads. cDNA data was compared with DNA, from kernels from the same batch (Table 1) sequenced in [26].

Endogenous nucleic acid content in HiSeq 2000 libraries (Table S3) was similar to that observed in the GS FLX libraries (Table S2) for both the cDNA and DNA, however the yield of uniquely mapped sequences was ∼200 times higher, allowing a more in-depth analysis of cDNA reads. HiSeq 2000 reads were visualized and contigs, defined as contiguous covered regions in the reference genome, were generated based on the cDNA library with the most reads (935130) using SeqMonk [30]. Additionally, we compared the percentage of reads that mapped to maize, in a non-maize sample (archaeological sunflower seed – results not shown), as to ensure that maize reads detected in our maize samples were not due to contamination occurring in the laboratory. For 23,615,527 indexed reads, we obtained 2711 (0.011%) maize reads, indicating that our high level of maize endogenous reads in both our DNA and RNA samples are not the product of contamination.

To evaluate the level of overlap between cDNA and DNA libraries, contig coordinates were then used to query the same regions in the mapped alignments for the two remaining samples and the read content per contig was recorded.

Using read content quantifications per contig, a Pearson's correlation matrix was calculated to compare cDNA and shotgun experiments (Table 2). A positive correlation was found for cDNA samples (0.8837) whereas negative correlations were found for both cDNA-shotgun comparisons (−0.0027 and −0.0099).

**Table 2:** Positive correlation matrix of contig read content from cDNA (935130 and 935230) and DNA [25].

	935130	935230	DNA
**935130**	1	0.08837647	−0.002722623
**935230**	0.8837647	1	−0.009936311

Finally, mapDamage [30] was used to visualize the fragmentation and misincorporation patterns of both cDNA and DNA libraries. The results of this analysis can be found in Figure S3, S4, and S5.

#### Identification of functional exon information

In order to extract potential functional information, B73 [27] exon annotations were overlapped with the position of reads. Most of the hits had no associated description; only few annotations with functional description were retrieved with more than three overlapping reads. The top 50 functionally annotated hits for sample 935130 is shown in Table 3 (Table S4) and the full list of exon hits with more than three reads can be found in Tables S5 and S6. The most commonly occurring gene hits were RING zinc finger protein-like, TMV response-related protein, heat shock protein 101 and multidrug resistance associated protein 1. All of these genes are linked with water deficiency and stress response; hence perhaps it is not surprising that we see these genes frequently.

**Table 3:** Top 50 exon hits with functional annotation from cDNA (935130 and 935230) HiSeq 2000 maize reads (for a detailed version of the table see Supplementary Table S4).

Sample	Reads	Exon Description
935130	3	cupin, RmlC-type
935130	3	Membrane protein
935130	3	AIR12
935130	3	Disease resistance gene analog PIC15 Fragment
935130	3	WRKY69 - superfamily of TFs having WRKY and zinc finger domains
935130	3	IQ calmodulin-binding motif family protein
935130	3	cytokinin-O-glucosyltransferase 1
935130	3	nitrate and chloride transporter
935130	3	invertase cell wall4 (incw4)
935130	5	anthranilate phosphoribosyltransferase-like protein
935130	3	inhibitor of apoptosis-like protein
935130	3	calmodulin-related protein 2, touch-induced
935130	3	meiosis 5
935230	3	amidophosphoribosyltransferase
935130	3	CDPK protein
935130	3	DNA binding protein
935130	3	WRKY71 - superfamily of TFs having WRKY and zinc finger domains
935130	3	60S ribosomal protein L19-3
935130	3	F-box domain containing protein
935130	3	MADS-box transcription factor 26
935230	3	RING zinc finger protein-like
935130	3	ring canal kelch
935130	3	plant-specific domain TIGR01568 family protein
935130	3	glycerol-3-phosphate acyltransferase 8
935130	3	ethanolaminephosphotransferase
935230	3	transmembrane BAX inhibitor motif-containing protein 4
935230	3	elongation factor Tu
935130	3	TMV response-related protein
935130	3	fasciclin-like arabinogalactan protein 8
935130	3	anther-specific proline-rich protein APG
935230	3	heat-shock protein 101
935230	3	CCCH transcription factor
935130	3	ubiquitin-protein ligase
935130	3	WRKY DNA-binding protein
935130	3	TMV response-related protein
935130	3	MTD1
935130	3	Nodulation signaling pathway 2 protein
935130	3	F-box protein
935130	3	cellulose synthase8
935130	4	metacaspase type II
935130	3	sialyltransferase-like protein
935130	3	3-methyl-2-oxobutanoate hydroxymethyltransferase
935130	3	indole-3-acetate beta-glucosyltransferase
935130	3	Serine threonine kinase
935130	4	glucan endo-1,3-beta-glucosidase 5
935130	3	sulfate transporter 3.4
935230	3	multidrug resistance associated protein 1
935230	3	hexose carrier protein HEX6
935130	3	beta-fructofuranosidase, insoluble isoenzyme 2
935130	3	invertase cell wall3

For the detection of exon-exon junctions, the totality of unmapped GS FLX reads were subjected to BLAT searches against B73 maize reference genome [27]. BLAT mapping allows a more accurate estimate of endogenous DNA in our samples since it permits split-read mapping, which is important in regions with genomic rearrangements, and necessary to find exon-exon junctions.

For the DNA (AZ Shotgun) from [26] and both HiSeq 2000 cDNA data sets, sequence duplicates were removed before BLAT search, to avoid redundancy as well as computational time. All reads with two hits in the same chromosome and strand were further analyzed in search of possible splice sites, however, no exon-exon junctions were found.

#### Repeat content analysis

Repeat content of sequences was explored using RepeatMasker [32] (RM database version 20110419) and repeat content profiles were compared between cDNA and DNA [26] samples. Sequences were retrieved from bam files where clones had been removed and paralogs were still present. Keeping paralogs was important since their removal would cause all the reads within the repeated regions to be excluded from the analysis. For the DNA sample [26] a subsample of 100,000 was randomly selected for this analysis. The dominant elements reported by the RepeatMasker from the two cDNA and one DNA sample can be found in Table 4 (Table S7 for unabridged version).

**Table 4:** Dominant elements represented in repeat content profiles of cDNA and DNA sequences using RepeatMasker.

Elements	No. of Elements			Length Occupied (bp)			Percentage of sequences (%)		
	DNA	935130	935230	DNA	935130	935230	DNA	935130	935230
Retroelements	45,689	57	886	1,941,117	37,987	2,637	47.88	0.05	0.01
Total interspersed repeats	47,989	65	926	2,037,874	39,479	3,347	50.26	0.05	0.01
Small RNA	224	459,802	988,699	9,710	64,585,935	25,848,184	0.24	89.04	72.42

As expected, both cDNA libraries showed a depletion of interspersed repeats and an enrichment of smallRNA elements, comprising mainly long and short subunits of rRNA, representing on average ∼80% of the non-clonal mapped reads (Table 4 and Table S7).

#### Taxonomic characterization of unmapped reads

**Figure 2:**
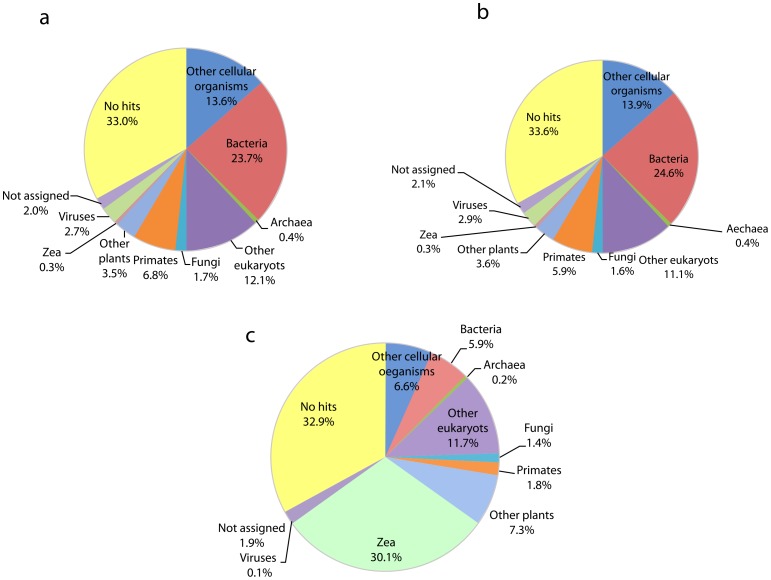
Taxonomic distribution of non-maize HiSeq 2000 reads from cDNA and DNA sets: (**a**) represents cDNA sample 935130, (**b**) represents cDNA sample 935230, and (**c**) represents a DNA shotgun sample from [25] 100,00 randomly sampled unmapped reads were used to perform BLAST searches and MEGAN was for taxonomic characterization of non-maize reference genome (non-B73) reads.

In order to explore the source of the non-maize sequences, 100,000 reads were randomly sampled from the unmapped reads from each sample and a BLAST search (-p blastn) [33] was performed against the nt database. BLAST outputs were then analyzed using MEGAN version .4.62.7 [34] with default parameters for the LCA-assignment algorithm. Approximately 35% of the reads fell in the unassigned or no hits category for all datasets. Among the assigned reads around a third corresponded to prokaryotes (bacteria and archaea) for the cDNA (Fig. 2a and b) and a much smaller fraction for DNA (Fig. 2c). The presence of fungal and primate hits, indicates some environmental and sampling contamination (Fig. 2). Furthermore, *Zea mays* sequences were also retrieved in the BLAST searches, most likely representing sequences not assembled into the reference B73 genome.

## Discussion

Several observations support the authenticity of the cDNA reads as being derived from ancient maize RNA (as opposed to undigested DNA or modern maize RNA), and that the content contains measurable levels of transcripts other than rRNA, thus are potentially useful for future paleotranscriptomic studies.

Firstly, the majority of the cDNA reads that matched maize were of ribosomal origin (∼80%), as shown by the repetitive elements analysis (based on Illumina data), which is in contrast to the rRNA content of the DNA library, where only 0.24% of the maize-matching reads were rRNA, likely reflecting a portion of rRNA gene content of the maize genome [35]. Moreover, depletion of interspersed repeats in cDNA libraries and enrichment in DNA, also support this observation.

Secondly, the taxonomic distribution of non-maize reads varies considerably when comparing the DNA [26] and the two cDNA samples (Fig. 2), whereas the cDNA samples are highly similar. Such difference is most likely explained by the distinct patterns of DNA and RNA survival of non-endogenous nucleic acid contamination. Furthermore, consistent with previous ancient genomic studies [36], the majority of the non-endogenous reads lacks a significant match to the nt database, a large proportion of which, may represent unsequenced environmental organisms or unassembled regions in the maize genome.

An unexpectedly large fraction of the taxonomic distribution corresponded to *Zea* (maize that has not been assembled to the B73 reference genome) in the DNA (Fig. 2c) set and but not in the RNA (Fig. 2a and b). In principle, this favors the notion that indeed RNA (from the cDNA samples), rather than DNA, was extracted from the maize kernels since functional and transcribed sequences are most likely to be conserved between B73 genome and other landraces, while random nuclear data is more prone to divergence and consequently, is unable to be mapped to the B73 genome. Alternatively, hits might also correspond to BAC clones not assembled into the maize reference genome but present in the nt database.

Thirdly, a positive correlation (Table 2) was found when comparing contig read content for cDNA samples whereas negative correlations were found for both cDNA-DNA comparisons implying an overlap of enriched regions in both cDNA experiments (Table 2) and suggesting a different nucleic acids source between cDNA and DNA libraries.

Lastly, fragmentation and misincorporation patterns of the cDNA (935130 and 935230) and DNA (AZ Shotgun) libraries, generated using mapDamage [31] (Figures S3, S4, and S5), were compared. For the DNA library, misincorporation as well as fragmentation plots clearly reflected patterns representative of aDNA: excess of purines (A and G) one genomic coordinate before the read, as well as C to T and G to A modifications towards the 5′ and 3′ ends of the reads [37]. In contrast, the cDNA libraries, while strikingly similar, showed none of the aforementioned features of the DNA sample. These results, therefore, imply that non-random mechanisms, different to those in DNA, are responsible for producing such patterns.

**Figure 3:**
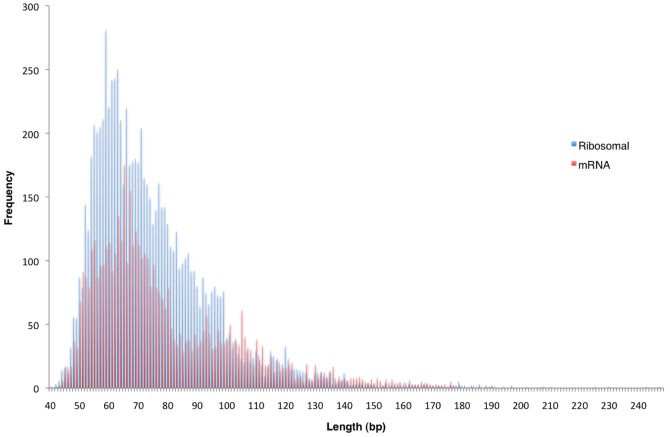
Histograms showing fragment length distribution of maize GS FLX sequence reads from cDNA samples (FLX1-3), comparing ribosomal RNA (red) to messenger RNA (blue) fragment lengths.

Further tests were performed to characterize the survival of our ancient maize RNA. Firstly, rRNA transcripts *in vivo* form secondary structures that result in large portions of the transcript existing as a double stranded molecule. A simple comparison of the mean, median and total read length distributions provides a simple means to investigate whether this double strandedness might confer a survival advantage of rRNA over other single stranded RNA transcripts (e.g. mRNAs). For GS FLX reads annotated as maize rRNA in a BLAST search, the mean and median lengths were 75.23 and 70 bp, respectively, while the lengths for mRNAs were 79.56 and 71 bp respectively. Thus, as visually demonstrated in Fig. 3, there appears to be no evidence of preferential survival of rRNA over mRNAs.

Additionally, a comparison of the maize DNA versus cDNA GS FLX sequence read lengths was made, showing that the modal read lengths for the cDNA averaged 64 bp compared to 54 bp for the DNA, suggesting that less fragmentation has occurred in RNA than DNA in these maize seeds (Fig. 1). Furthermore, when the modes of rRNA and mRNA were compared from all cDNA samples the modal mRNA length is higher than the modal rRNA value. These results correspond to the requirement for long-term mRNA survival in seeds [19], [38]–[40], and are promising for future studies that might aim to sequence RNA from seeds dating back to the early phases of domestication.

In summary, we demonstrate the long-term survival of, and ability to RNA-Seq, RNA sequences of informative length in archaeological maize kernels. The long-term survival of RNA could be due to the presence of biological mechanisms in seeds for RNA preservation or, as suggested by Venanzi and Rollo [22], may simply reflect the relative level of RNA over DNA. Dormant plant seeds contain mRNA that was transcribed during late embryogenesis and is translated during germination [38]–[40] thus it is perhaps not surprising that mRNA can be detected. However, further research will be required, involving vastly greater sample and data sets, to be able to determine the accuracy of ancient gene expression studies. Furthermore, the authors acknowledge that even if relative abundances of RNA reads reflect transcription when the plant was alive, it would be difficult to compare an ancient seed in one environment to a modern seed in a different environment. Hence, further research would also be required to explore if multiple seeds show similar patterns of transcript abundance that could not otherwise be explained by differences in relative survival compared to modern corn in various environmental settings.

Given (i) the generation here of diverse transcriptomic sequences, (ii) previous documentation of nucleic acid survival in seeds spanning back thousands of years, (iii) the large amounts of well-preserved ancient crop seeds that are held in archaeological collections, often spanning the temporal and geographic range of the species' domestication history, and (iv) the importance of transcriptomal modification during domestication, we suggest that the future study of ancient transcriptomics from dried seeds may offer a powerful new tool with which to complement our understanding of crop domestication.

## Supporting Information

Supplementary Methods S1 **Detailed methodology, including nucleic acid extraction method, PCR amplification primer sequences and tests, data analyses and radiocarbon dating.**
(DOCX)

Figure S1 **Photo of Arizona kernel, batch 935.**
(TIF)

Figure S2 **Calibration curves for dating of 2 Arizona maize kernels.**
(TIF)

Figure S3 **Ancient DNA (AZ Shotgun) Fragmentation and Misincorporation Plot.**
(TIF)

Figure S4 **Ancient RNA (935130) Fragmentation and Misincorporation Plot.**
(TIF)

Figure S5 **Ancient RNA (935230) Fragmentation and Misincorporation Plot.**
(TIF)

Table S1 **Radiocarbon results BP and analytical data, including stable isotope results.** All data is acceptable for a material such as this. ‘Used’ is the material analyzed in pretreatment chemistry, whilst ‘yield’ is the amount remaining after the chemical purification procedures applied.
(DOCX)

Table S2 **Fraction of total GS FLX reads mapping to the B73 reference genome for cDNA (4) and DNA (3) libraries.** The breakdown of reads mapped with BWA before and after removing sequence duplicates and paralogs is shown. Unmapped reads were then mapped using BLAT to retrieve as many endogenous reads as possible. Estimates of endogenous maize nucleic acid content are highlighted in bold.
(DOCX)

Table S3 **Fraction of total GAIIx (AZ shotgun) and HiSeq (935130 and 935230) reads mapped to the B73 reference genome.** BWA (before and after removing sequence duplicates and paralogs) and BLAT mapping values are shown.
(DOCX)

Table S4 **Top 50 exon hits with functional annotation from 935130 cDNA maize read.**
(DOCX)

Table S5 **Functionally annotated exon hits for Arizonan kernel 935130.**
(DOCX)

Table S6 **Functionally annotated exon hits for Arizonan kernel 935230.**
(DOCX)

Table S7 **Repeat content profiles of HiSeq cDNA and GAIIx DNA sequences using RepeatMasker.**
(DOCX)
