# Supplementary material for: Deep Sequencing of RNA from Ancient Maize Kernels
Source: PLoS One. 2013 Jan 11;8(1):e50961. doi: 10.1371/journal.pone.0050961 (PMC3543400; doi:10.1371/journal.pone.0050961)
Supplement: Supplementary Methods S1 — Detailed methodology, including nucleic acid extraction method, PCR amplification primer sequences and tests, data analyses and radiocarbon dating. (DOCX) [file pone.0050961.s001.docx]

**Deep sequencing of RNA from ancient maize kernels**

**Supplementary Methods**

**Materials and Methods**

**Nucleic Acid Extractions**

Manipulation of the ancient samples was undertaken in a dedicated ancient DNA laboratory to minimize the chance of extracting contamination from external sources of nucleic acids. We note, that given the nature of the study (that is, the result generation not being based on amplicons), conventional contamination challenges are unlikely to affect the data. In order to minimize the level of exogenous material in the samples, and to limit the possibility that RNA in the chosen seeds had been compromised by exogenous RNases, where possible, the seeds sampled were chosen based on the structural integrity of the testa. We furthermore hypothesized that samples with an intact testa (e.g. Fig. S1) could be bathed in 10% solution of sodium hypochlorite solution prior to extraction, without compromising the internal nucleic acids. This method has been previously demonstrated a suitable means of reducing contaminant DNA sequences present on the outside of other relatively impermeable ancient materials, such as hair and nail (S1, S2). Kernels were then rinsed in certified DNA-free molecular biology grade water. Subsequently, dried kernels were ground to a coarse powder.

A 750μl volume of lysis buffer (10mM Tris-HCl pH8.0, 10mM sodium chloride, 2% w/v sodium dodecyl sulfate (SDS), 5mM calcium chloride, 2.5mM ethylene-diamine-tetra-acetic acid pH8.0, 40mM dithiothreitol, 10% proteinase K solution) was added to each kernel, vortexed thoroughly and incubated overnight at 55°C.

Following the incubation period, DNA and RNA were co-extracted from the digest using a standard sequential organic extraction (1:1:1 volumes of phenol, phenol, then chloroform). Post chloroform extraction, both DNA and RNA were isolated from the final aqueous layer using a QIAquick purification kit (Qiagen, Hilden, Germany), following the manufacturer’s protocol, with final nucleic acid elution into 40µl of EB. In general, 20µl of this elute was subjected to DNA analysis while the remaining 20µl was subjected to cDNA synthesis.

**PCR Amplification**

PCR tests were as followed:

1) PCR using primer set *Rbcl-DNA* (Forward: 5’ – TTCATGAGTTGTAGGGAGGGAC, reverse: 5’ – TTCGTACTCCGGGGTGTAGT, amplicon size 115bp) was performed. *Rbcl-DNA* is a primer set that was designed to target a DNA fragment that sits over the border of an exon and intron within the plant chloroplast genome. As RNA transcripts lack the intronic part of the sequence, they are un-amplifiable using this primer set, thus only DNA can be amplified. The region targeted is conserved among plants in general, thus this single primer set should theoretically work on a wide range of target species. This primer was used as a control to initially check DNA was in the original extracts, and then later check that DNA was not in the DNase treated, cDNA extracts.

2) PCR using primer set *Rbcl* (Forward: 5’ GGCAGCATTCCGAGTAACTCCTC, reverse: 5’ CGTCCTTTGTAACGATCAAG, amplicon size 140bp, (S3)) was performed. This primer set is a generic set that sits within an exon of the Ribulose-biphospate carboxylase gene, and thus can amplify both DNA and cDNA from many plants, and thus can be used to indicate successful cDNA synthesis (thus RNA survival), under the condition that such cDNA does not also yield a PCR product using primer set *Rbcl-DNA* (above).

3) PCR using primer set *MLP1* (Forward: 5’ – CGAGGTCGAGAGAGAGAGGA, reverse: 5’ – ATGTCCGGGTACATCTTGCT, amplicon size 108bp) was performed. This primer set, designed to target cDNA of the maize-specific Metallothionein-like protein 1, is placed over an exon-exon boundary, and will only yield cDNA derived from maize RNA, thus is not susceptible to contamination problems due to cDNA from other plants.

All PCR reactions were undertaken in 25µl volumes using AmpliTaq Gold (Applied Biosystems, Foster City, CA). Each reaction contained 1X AmpliTaq Gold buffer, 2.5mM MgCl_2,_ 0.5µM forward primer, 0.5µM reverse primer, 0.2mM BSA, 0.2mM each dNTP, 0.1 µl enzyme and 1µl DNA or cDNA. The PCR cycling conditions were as follows: 95°C for 4’, 45 cycles of (95°C for 40”, 55°C for 35”, 72°C for 40”) and a final extension at 72°C for 10’.

Only cDNA samples that could be amplified with the *Rbcl* and *MLP1* primers were sequenced; whilst cDNA samples that were amplified with *Rbcl-DNA* primers were excluded, as they were likely to contain DNA.

**Alignment visualization, contig generation and annotation overlap**

Read alignments, in bam format, were visualized using SeqMonk version 0.20. To identify overrepresented regions in the cDNA libraries when compared to DNA libraries, contigs were generated for the library with the higher number of mapped reads (935130) and the same regions were queried in both remaining cDNA and DNA alignments to record the read content. A total of 46,042 contigs were generated.

Because the vast majority of the contigs contained only one read, a filter was set to consider only the top 1% of the read content distribution in both cDNA sets. This filter yielded a total of 335 contigs. Contigs were annotated based on the overlap they had with mRNA and common repeats (<http://ftp.maizesequence.org/current/repeats/>). Only 33 of the filtered contigs overlapped with annotated mRNAs, however, no functional description existed for any of these matches. When contigs were overlapped with repeat annotations 262 had some overlap with an annotated repeat, 254 corresponded to retrotransposon family ipiki and 8 to the yreud family.

For functional annotation, read positions were overlapped with B73 exon annotations. For GS FLX data only two reads had overlap with two exons (B4FUR6_MAIZE and B7X11_MAIZE), and for HiSeq datasets most of the hits had no associated description (Tables S5 and S6).

**Radiocarbon dating**

AMS carbon dating of 2 of the kernels was performed at the NERC ^14^C dating facility, University of Oxford, following standard protocols. The kernels, given the Oxford Accession numbers (OxA-21234 and OxA-21235) were dated as 707±23 and 733±23 ^14^CYBP respectively (Fig. S2, Table S1).

AMS radiocarbon was undertaken at the Oxford Radiocarbon Accelerator Unit (ORAU), University of Oxford. The seed samples were treated with a routine acid-base-acid treatment (A-B-A) consisting of a wash in 1 M HCl at RT, a NaOH wash at 80°C for one hour, and a third 1 M HCl wash. The samples were rinsed between steps using double distilled water. Finally, the samples were lightly bleached using 2.5% (w:vol) NaClO_2_ solution adjusted to pH 3 for ~30 mins (see S4 for more detail of the ORAU preparative methods).

Pre-treated samples were combusted and analysed using a Europa Scientific elemental analyser system coupled to a Europa Geo 20-20 IR mass spectrometer operating in continuous flow mode, using an He carrier gas. δ^13^C values are reported with reference to VPDB. The samples were graphitised by reduction of CO_2_ over an iron catalyst in an excess H_2_ atmosphere at 560°C (S5) and dated using the HVEE AMS in Oxford (S6). The radiocarbon dating results were calibrated using the INTCAL04 curve of S7 and the OxCal software (S8). (Fig. S2). The results (Table S1) are consistent with the 13^th^ century AD.

**Supplementary References**

(S1) Gilbert, M.T., Bandelt, H.J., Hofreiter, M. & Barnes, I. Assessing ancient DNA studies. *Trends in ecology & evolution* **20**, 541-544 (2005).

(S2) Gilbert, M.T. et al. Whole-genome shotgun sequencing of mitochondria from ancient hair shafts. *Science* **317**, 1927-1930 (2007).

(S3) Poinar, H.N. et al. Molecular coproscopy: dung and diet of the extinct ground sloth Nothrotheriops shastensis. *Science* **281**, 402-406 (1998).

(S4) Brock *et al.,* Current pretreatment methods for AMS radiocarbon dating at the Oxford Radiocarbon Accelerator Unit (ORAU). *Radiocarbon* **52**, 1 (2010).

(S5) Dee, M. and Bronk Ramsey, C. Refinement of graphite target production at ORAU. *Nucl. Instr. and Meth. B,* **172** (2000).

(S6) Bronk Ramsey, C., Ditchfield, P., Humm, M. *et al.,* Using a Gas ion source for radiocarbon AMS and GC-AMS. *Radiocarbon* **46,** 1 (2004).

(S7) Reimer *et al.,* IntCal04 terrestrial radiocarbon age calibration, 0-26 cal kyr BP. *Radiocarbon* **46**, 3 (2004).

(S8) Bronk Ramsey, C. Development of the radiocarbon calibration program OxCal. *Radiocarbon* **43**, 2A (2001).
