# Supplementary figures and images for: Deep Sequencing of RNA from Ancient Maize Kernels
Source: PLoS One. 2013 Jan 11;8(1):e50961. doi: 10.1371/journal.pone.0050961 (PMC3543400; doi:10.1371/journal.pone.0050961)

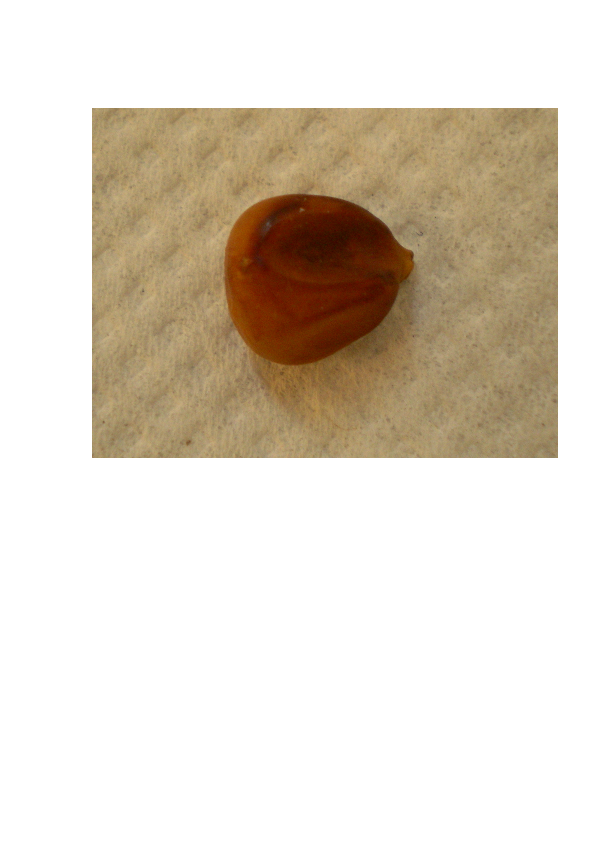

Supplement: Figure S1 — Photo of Arizona kernel, batch 935. (TIF) [file pone.0050961.s002.tif]

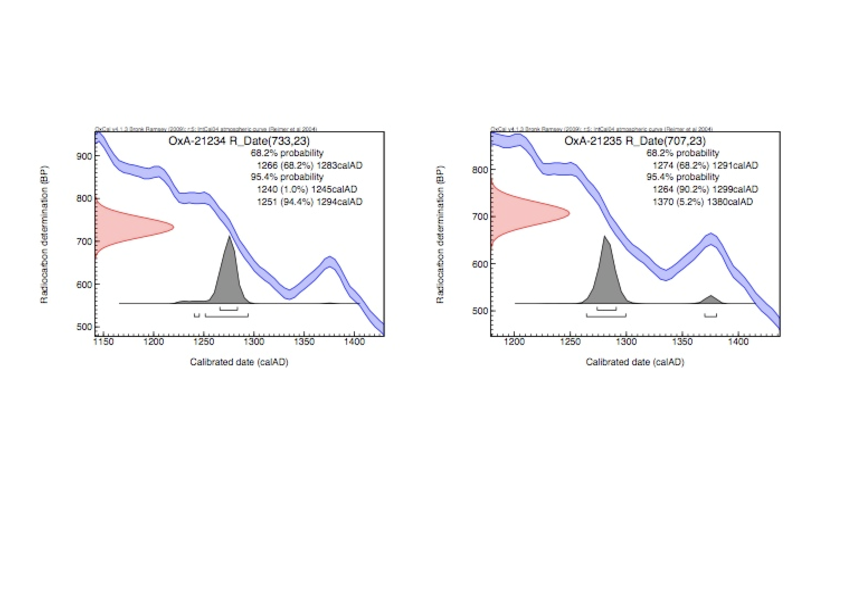

Supplement: Figure S2 — Calibration curves for dating of 2 Arizona maize kernels. (TIF) [file pone.0050961.s003.tif]

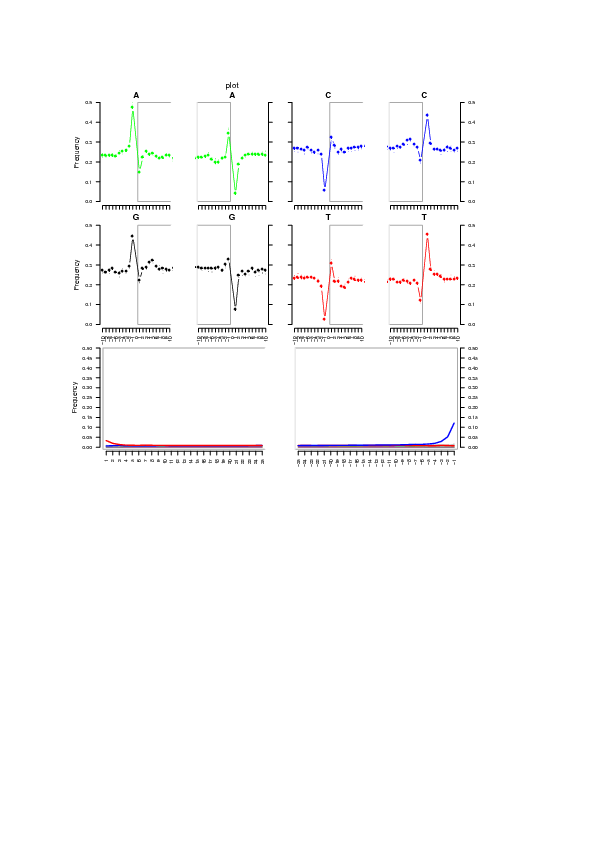

Supplement: Figure S3 — Ancient DNA (AZ Shotgun) Fragmentation and Misincorporation Plot. (TIF) [file pone.0050961.s004.tif]

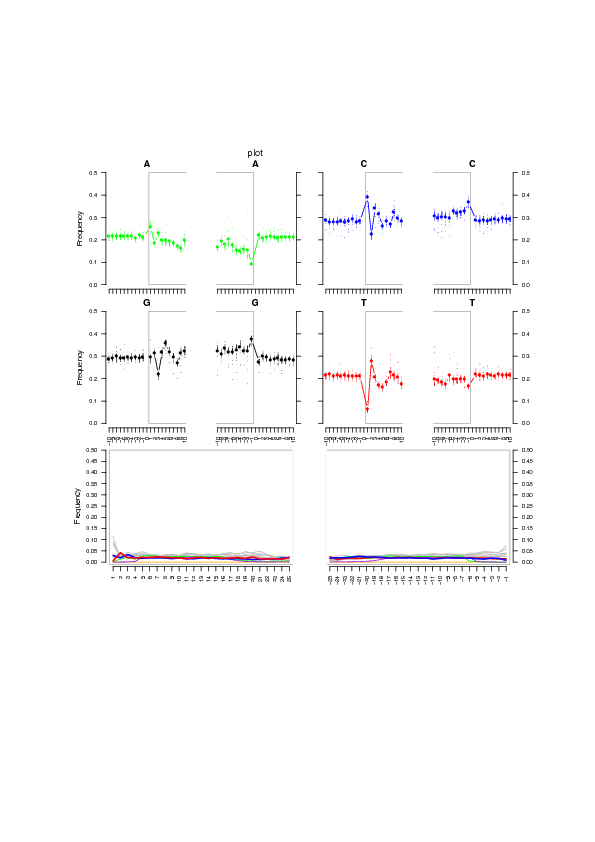

Supplement: Figure S4 — Ancient RNA (935130) Fragmentation and Misincorporation Plot. (TIF) [file pone.0050961.s005.tif]

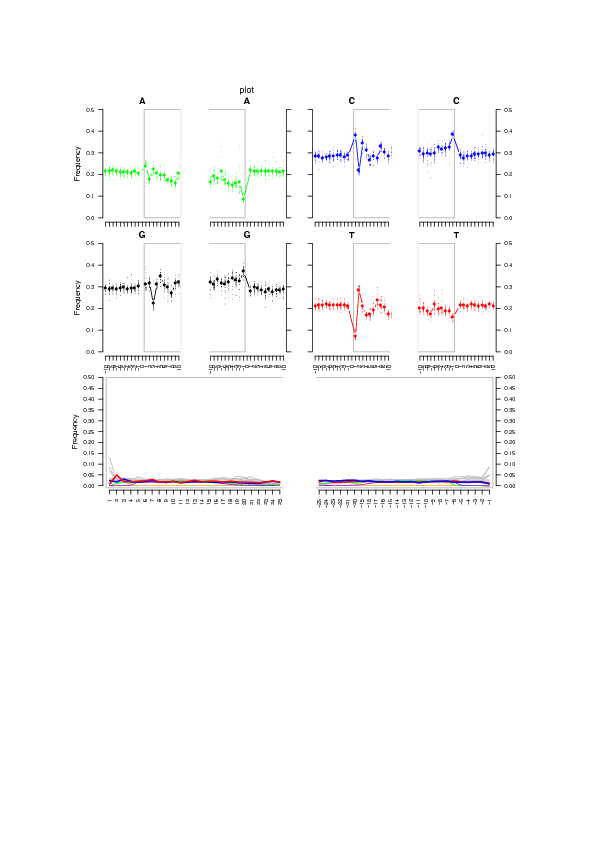

Supplement: Figure S5 — Ancient RNA (935230) Fragmentation and Misincorporation Plot. (TIF) [file pone.0050961.s006.tif]
