# Supplementary material for: Deep Sequencing of RNA from Ancient Maize Kernels
Source: PLoS One. 2013 Jan 11;8(1):e50961. doi: 10.1371/journal.pone.0050961 (PMC3543400; doi:10.1371/journal.pone.0050961)
Supplement: Table S1 — Radiocarbon results BP and analytical data, including stable isotope results. All data is acceptable for a material such as this. ‘Used’ is the material analyzed in pretreatment chemistry, whilst ‘yield’ is the amount remaining after the chemical purification procedures applied. (DOCX) [file pone.0050961.s007.docx]

**Table S1**

| **OxA** | **Radiocarbon date BP** | **Used (mg)** | **Yield (mg)** | **%Yld** | **%C** | **d13C** |
| --- | --- | --- | --- | --- | --- | --- |
| 21234 | 733 ± 23 | 153.97 | 61.03 | 39.6 | 41.8 | -9.8 |
| 21235 | 707 ± 23 | 78.26 | 27.09 | 34.6 | 40.6 | -8.5 |
