# Supplementary material for: Deep Sequencing of RNA from Ancient Maize Kernels
Source: PLoS One. 2013 Jan 11;8(1):e50961. doi: 10.1371/journal.pone.0050961 (PMC3543400; doi:10.1371/journal.pone.0050961)
Supplement: Table S2 — Fraction of total GS FLX reads mapping to the B73 reference genome for cDNA (4) and DNA (3) libraries. The breakdown of reads mapped with BWA before and after removing sequence duplicates and paralogs is shown. Unmapped reads were then mapped using BLAT to retrieve as many endogenous reads as possible. Estimates of endogenous maize nucleic acid content are highlighted in bold. (DOCX) [file pone.0050961.s008.docx]

**Table S2**

|  |  | **cDNA** |  |  | **DNA** |  |
| --- | --- | --- | --- | --- | --- | --- |
|  | **FLX1** | **FLX2** | **FLX3** | **FLX4** | **FLX5** | **FLX6** |
| **total reads** | 9944 | 4494 | 7900 | 39945 | 37558 | 7199 |
| **mapped to maize** | 4730 | 3061 | 5622 | 25283 | 25872 | 3036 |
| **%** | 47.56637168 | 68.11303961 | 71.16455696 | 63.29452998 | 68.88545716 | 42.17252396 |
| **without duplicates** | 4072 | 2520 | 4702 | 21690 | 22461 | 2189 |
| **uniquely** | 271 | 177 | 203 | 2718 | 3216 | 380 |
| **Unmapped** | 5214 | 1433 | 2278 | 14662 | 11686 | 4163 |
| **Mapped with BLAT** | 3040 | 898 | 1514 | 2676 | 9815 | 3963 |
| **Non-maize** | 2174 | 535 | 764 | 11986 | 1871 | 200 |
|  |  | **Total Reads cDNA** | **22338** |  | **Total Reads DNA** | **84702** |
