# Supplementary material for: Deep Sequencing of RNA from Ancient Maize Kernels
Source: PLoS One. 2013 Jan 11;8(1):e50961. doi: 10.1371/journal.pone.0050961 (PMC3543400; doi:10.1371/journal.pone.0050961)
Supplement: Table S3 — Fraction of total GAIIx (AZ shotgun) and HiSeq (935130 and 935230) reads mapped to the B73 reference genome. BWA (before and after removing sequence duplicates and paralogs) and BLAT mapping values are shown. (DOCX) [file pone.0050961.s009.docx]

**Table S3**

|  | **AZ shotgun** | **935130** | **935230** |
| --- | --- | --- | --- |
| **Total reads** | 38836914 | 147443152 | 112672928 |
| **Trimmed** | 38570708 | 111404091 | 95676534 |
| **Mapped to Maize** | 35630554 | 74791427 | 73628151 |
| **%Maize** | **0.923772361** | **0.671352608** | **0.769552867** |
| **Clonal removal** | 33304905 | 932707 | 1299962 |
| **Unique/good quality** | 8173084 | 52713 | 34452 |
| **Unmapped** | 2940154 | 36612664 | 22048383 |
| **unmapped rmdup** | 2317247 | 5591360 | 3147820 |
| **Blat mapped** | 805960 | 496076 | 427393 |
| **Non-maize** | 1511228 | 5095284 | 2720427 |
