# Supplementary material for: Deep Sequencing of RNA from Ancient Maize Kernels
Source: PLoS One. 2013 Jan 11;8(1):e50961. doi: 10.1371/journal.pone.0050961 (PMC3543400; doi:10.1371/journal.pone.0050961)
Supplement: Table S4 — Top 50 exon hits with functional annotation from 935130 cDNA maize read. (DOCX) [file pone.0050961.s010.docx]

| **Sample** | **Chr** | **Start** | **End** | **Reads** | **ID** | **Source** | **Accession No.** | **Description** |
| --- | --- | --- | --- | --- | --- | --- | --- | --- |
| 935130 | 1 | 182292591 | 182293471 | 3 | B6SMC5_MAIZE | RefSeq Peptide | NP_001147194 | cupin, RmlC-type |
| 935130 | 1 | 191859884 | 191861906 | 3 | B6UA62_MAIZE | UniProtKB/TrEMBL | B6UA62 | Membrane protein |
| 935130 | 1 | 191893507 | 191894902 | 3 | B6SN55_MAIZE | RefSeq Peptide | NP_001147237 | AIR12 |
| 935130 | 1 | 196691877 | 196695603 | 3 | Q9ZTJ0_MAIZE | UniProtKB/TrEMBL | Q9ZTJ0 | Disease resistance gene analog PIC15 Fragment |
| 935130 | 1 | 216580897 | 216582541 | 3 | B6TTZ5_MAIZE | RefSeq Peptide | NP_001150829 | WRKY69 - superfamily of TFs having WRKY and zinc finger domains |
| 935130 | 1 | 253134377 | 253135475 | 3 | B6SSD5_MAIZE | RefSeq Peptide | NP_001147510 | IQ calmodulin-binding motif family protein |
| 935130 | 1 | 275582841 | 275584525 | 3 | B6THX4_MAIZE | RefSeq Peptide | NP_001149762 | cytokinin-O-glucosyltransferase 1 |
| 935130 | 2 | 232648880 | 232651058 | 3 | B6U7W3_MAIZE | RefSeq Peptide | NP_001152028 | nitrate and chloride transporter |
| 935130 | 2 | 3188143 | 3188640 | 3 | Q9SBI5_MAIZE | RefSeq DNA | NM_001111429 | invertase cell wall4 (incw4) |
| 935130 | 2 | 38562586 | 38565546 | 5 | C0PCM4_MAIZE | RefSeq Peptide | NP_001148072 | anthranilate phosphoribosyltransferase-like protein |
| 935130 | 2 | 60874151 | 60875855 | 3 | B6STV7_MAIZE | RefSeq Peptide | NP_001147662 | inhibitor of apoptosis-like protein |
| 935130 | 3 | 20704046 | 20705189 | 3 | B6SRF1_MAIZE | RefSeq Peptide | NP_001147546 | calmodulin-related protein 2, touch-induced |
| 935130 | 3 | 4843609 | 4844855 | 3 | B6TNA8_MAIZE | RefSeq Peptide | NP_001150305 | meiosis 5 |
| 935230 | 3 | 173801386 | 173803474 | 3 | B6SRU6_MAIZE | RefSeq Peptide | NP_001147451 | amidophosphoribosyltransferase |
| 935130 | 4 | 168737104 | 168738634 | 3 | C3UZ62_MAIZE | RefSeq Peptide | NP_001170479 | CDPK protein |
| 935130 | 4 | 226067153 | 226068764 | 3 | B6SJB5_MAIZE | RefSeq Peptide | NP_001146992 | DNA binding protein |
| 935130 | 4 | 235118646 | 235119794 | 3 | GRMZM2G036711 | RefSeq DNA | NM_001154260 | WRKY71 - superfamily of TFs having WRKY and zinc finger domains |
| 935130 | 4 | 237311806 | 237314176 | 3 | B6T208_MAIZE | RefSeq Peptide | NP_001150484 | 60S ribosomal protein L19-3 |
| 935130 | 4 | 26653585 | 26655098 | 3 | B6U9N7_MAIZE | RefSeq Peptide | NP_001152150 | F-box domain containing protein |
| 935130 | 4 | 38758715 | 38760499 | 3 | C4J2F4_MAIZE | RefSeq Peptide | NP_001148873 | MADS-box transcription factor 26 |
| 935230 | 4 | 155910424 | 155911806 | 3 | B6SMS1_MAIZE | RefSeq Peptide | NP_001147218 | RING zinc finger protein-like |
| 935130 | 5 | 112753088 | 112754513 | 3 | B6TP81_MAIZE | RefSeq Peptide | NP_001150390 | ring canal kelch |
| 935130 | 5 | 196249840 | 196251589 | 3 | B4FVC0_MAIZE | RefSeq Peptide | NP_001148277 | plant-specific domain TIGR01568 family protein |
| 935130 | 5 | 69114970 | 69116904 | 3 | B6SRR0_MAIZE | RefSeq Peptide | NP_001147442 | glycerol-3-phosphate acyltransferase 8 |
| 935130 | 5 | 70772846 | 70776203 | 3 | B6U6I8_MAIZE | RefSeq Peptide | NP_001151915 | Ethanolaminephosphotransferase |
| 935230 | 5 | 7594371 | 7595057 | 3 | B6TZF1_MAIZE | RefSeq Peptide | NP_001151352 | transmembrane BAX inhibitor motif-containing protein 4 |
| 935230 | 5 | 180632305 | 180634311 | 3 | B6TPG0_MAIZE | RefSeq Peptide | NP_001150410 | elongation factor Tu |
| 935130 | 6 | 114571949 | 114573320 | 3 | B6UBT3_MAIZE | RefSeq Peptide | NP_001152287 | TMV response-related protein |
| 935130 | 6 | 124803945 | 124805261 | 3 | C0P928_MAIZE | RefSeq Peptide | NP_001147121 | fasciclin-like arabinogalactan protein 8 |
| 935130 | 6 | 147690396 | 147690950 | 3 | B6SUN1_MAIZE | RefSeq Peptide | NP_001147753 | anther-specific proline-rich protein APG |
| 935230 | 6 | 160621048 | 160622526 | 3 | C0PDC7_MAIZE | RefSeq Peptide | NP_001104935 | heat-shock protein 101 |
| 935230 | 6 | 160013893 | 160015192 | 3 | B6SLI7_MAIZE | RefSeq Peptide | NP_001147375 | CCCH transcription factor |
| 935130 | 7 | 103876337 | 103878886 | 3 | B6SWJ0_MAIZE | RefSeq Peptide | NP_001147953 | ubiquitin-protein ligase |
| 935130 | 7 | 115309658 | 115310466 | 3 | B1Q040_MAIZE | RefSeq Peptide | NP_001120723 | WRKY DNA-binding protein |
| 935130 | 7 | 134038900 | 134039768 | 3 | B6SUL7_MAIZE | RefSeq Peptide | NP_001147747 | TMV response-related protein |
| 935130 | 7 | 137824231 | 137825672 | 3 | B6T7R4_MAIZE | RefSeq Peptide | NP_001148854 | MTD1 |
| 935130 | 7 | 158368564 | 158370335 | 3 | B6SU23_MAIZE | UniProtKB/TrEMBL | B6SU23 | Nodulation signaling pathway 2 protein |
| 935130 | 7 | 165744759 | 165746434 | 3 | Q5GAU2_MAIZE | UniProtKB/TrEMBL | Q5GAU2 | F-box protein |
| 935130 | 7 | 26487882 | 26488851 | 3 | Q9LLI2_MAIZE | RefSeq Peptide | NP_001104958 | cellulose synthase8 |
| 935130 | 8 | 120777110 | 120778906 | 4 | B6U746_MAIZE | RefSeq Peptide | NP_001151968 | metacaspase type II |
| 935130 | 8 | 166836625 | 166838331 | 3 | B6U5U9_MAIZE | RefSeq Peptide | NP_001151877 | sialyltransferase-like protein |
| 935130 | 8 | 27215345 | 27216282 | 3 | B4FTG2_MAIZE | RefSeq Peptide | NP_001150103 | 3-methyl-2-oxobutanoate hydroxymethyltransferase |
| 935130 | 8 | 65981741 | 65983349 | 3 | B6UFB5_MAIZE | RefSeq Peptide | NP_001152529 | indole-3-acetate beta-glucosyltransferase |
| 935130 | 9 | 11777826 | 11780181 | 3 | Q8W1D3_MAIZE | UniProtKB/TrEMBL | Q8W1D3 | Serine threonine kinase |
| 935130 | 9 | 121937248 | 121938756 | 4 | B6SWD3_MAIZE | RefSeq Peptide | NP_001147933 | glucan endo-1,3-beta-glucosidase 5 |
| 935130 | 9 | 20337422 | 20338112 | 3 | B6SYY9_MAIZE | RefSeq Peptide | NP_001148179 | sulfate transporter 3.4 |
| 935230 | 9 | 57011529 | 57013736 | 3 | Q6Y3I1_MAIZE | RefSeq Peptide | NP_001105942 | multidrug resistance associated protein 1 |
| 935230 | 9 | 20747300 | 20749031 | 3 | B4FVI7_MAIZE | RefSeq Peptide | NP_001148202 | hexose carrier protein HEX6 |
| 935130 | 10 | 114237661 | 114239362 | 3 | B6U1H5_MAIZE | RefSeq Peptide | NP_001151535 | beta-fructofuranosidase, insoluble isoenzyme 2 |
| 935130 | 10 | 114289641 | 114289799 | 3 | Q9ZTQ5_MAIZE | RefSeq Peptide | NP_001104898 | invertase cell wall3 |

**Table S4**
