# Supplementary material for: Deep Sequencing of RNA from Ancient Maize Kernels
Source: PLoS One. 2013 Jan 11;8(1):e50961. doi: 10.1371/journal.pone.0050961 (PMC3543400; doi:10.1371/journal.pone.0050961)
Supplement: Table S5 — Functionally annotated exon hits for Arizonan kernel 935130. (DOCX) [file pone.0050961.s011.docx]

**Table S5**

| Chr | Start | End | Reads | ID | Description |
| --- | --- | --- | --- | --- | --- |
| 1 | 168908705 | 168909014 | 64 | GRMZM2G052361 | No description |
| 1 | 51463393 | 51463721 | 46 | GRMZM2G177990 | No description |
| 1 | 161046218 | 161046528 | 23 | GRMZM2G130258 | No description |
| 1 | 166323758 | 166324039 | 19 | GRMZM2G427402 | No description |
| 1 | 176412407 | 176412500 | 9 | GRMZM2G095389 | No description |
| 1 | 166323451 | 166323614 | 8 | GRMZM2G427402 | No description |
| 1 | 1779988 | 1782907 | 5 | GRMZM2G161540 | hypothetical protein LOC100381635 (LOC100381635), mRNA [Source:RefSeq DNA;Acc:NM_001174451] |
| 1 | 174227500 | 174229713 | 5 | GRMZM2G047875 | No description |
| 1 | 67771164 | 67773953 | 4 | B8A2U2_MAIZE | hypothetical protein LOC100280265 [Source:RefSeq peptide;Acc:NP_001146665] |
| 1 | 297304684 | 297305399 | 4 | B8A356_MAIZE | hypothetical protein LOC100280315 [Source:RefSeq peptide;Acc:NP_001146713] |
| 1 | 238564969 | 238567467 | 4 | B4G1L2_MAIZE | hypothetical protein LOC100274519 (LOC100274519), mRNA [Source:RefSeq DNA;Acc:NM_001148876] |
| 1 | 93909748 | 93911493 | 3 | GRMZM2G392477 | No description |
| 1 | 60776350 | 60777307 | 3 | GRMZM2G041803 | No description |
| 1 | 299405007 | 299407573 | 3 | B4F8M2_MAIZE | hypothetical protein LOC100191385 [Source:RefSeq peptide;Acc:NP_001130291] |
| 1 | 298156492 | 298158134 | 3 | B4F9D8_MAIZE | hypothetical protein LOC100274299 [Source:RefSeq peptide;Acc:NP_001142135] |
| 1 | 2904434 | 2907015 | 3 | GRMZM2G004798 | No description |
| 1 | 279898912 | 279900155 | 3 | C0PKN8_MAIZE | hypothetical protein LOC100384002 [Source:RefSeq peptide;Acc:NP_001170084] |
| 1 | 279185068 | 279187876 | 3 | B6U0R8_MAIZE | LOC100285096 [Source:RefSeq peptide;Acc:NP_001151463] |
| 1 | 275582841 | 275584525 | 3 | B6THX4_MAIZE | cytokinin-O-glucosyltransferase 1 [Source:RefSeq peptide;Acc:NP_001149762] |
| 1 | 2753595 | 2755628 | 3 | GRMZM2G086269 | No description |
| 1 | 253134377 | 253135475 | 3 | B6SSD5_MAIZE | IQ calmodulin-binding motif family protein [Source:RefSeq peptide;Acc:NP_001147510] |
| 1 | 246699300 | 246699563 | 3 | GRMZM2G447145 | No description |
| 1 | 234900778 | 234902766 | 3 | GRMZM2G075492 | No description |
| 1 | 22437817 | 22440310 | 3 | B6TRW7_MAIZE | LOC100284268 [Source:RefSeq peptide;Acc:NP_001150635] |
| 1 | 222400698 | 222402399 | 3 | GRMZM2G075247 | No description |
| 1 | 216580897 | 216582541 | 3 | B6TTZ5_MAIZE | WRKY69 - superfamily of TFs having WRKY and zinc finger domains [Source:RefSeq peptide;Acc:NP_001150829] |
| 1 | 196691877 | 196695603 | 3 | Q9ZTJ0_MAIZE | Disease resistance gene analog PIC15 Fragment [Source:UniProtKB/TrEMBL;Acc:Q9ZTJ0] |
| 1 | 191893507 | 191894902 | 3 | B6SN55_MAIZE | AIR12 [Source:RefSeq peptide;Acc:NP_001147237] |
| 1 | 191859884 | 191861906 | 3 | B6UA62_MAIZE | Membrane protein [Source:UniProtKB/TrEMBL;Acc:B6UA62] |
| 1 | 182292591 | 182293471 | 3 | B6SMC5_MAIZE | cupin, RmlC-type [Source:RefSeq peptide;Acc:NP_001147194] |
| 1 | 174996277 | 174997566 | 3 | GRMZM2G091278 | No description |
| 1 | 105404133 | 105404222 | 3 | GRMZM5G858771 | No description |
| 2 | 14313150 | 14313434 | 32 | GRMZM2G070188 | No description |
| 2 | 48392981 | 48393259 | 29 | GRMZM2G112649 | No description |
| 2 | 38562586 | 38565546 | 5 | C0PCM4_MAIZE | anthranilate phosphoribosyltransferase-like protein [Source:RefSeq peptide;Acc:NP_001148072] |
| 2 | 109105749 | 109108727 | 4 | GRMZM2G167658 | No description |
| 2 | 9435142 | 9436240 | 3 | GRMZM2G380518 | No description |
| 2 | 60874151 | 60875855 | 3 | B6STV7_MAIZE | inhibitor of apoptosis-like protein [Source:RefSeq peptide;Acc:NP_001147662] |
| 2 | 57126483 | 57128025 | 3 | C0PNA5_MAIZE | hypothetical protein LOC100384311 [Source:RefSeq peptide;Acc:NP_001170337] |
| 2 | 5214057 | 5215827 | 3 | GRMZM2G160523 | No description |
| 2 | 44697893 | 44698753 | 3 | GRMZM2G474783 | No description |
| 2 | 44199862 | 44202611 | 3 | B4FV90_MAIZE | hypothetical protein LOC100273399 [Source:RefSeq peptide;Acc:NP_001141308] |
| 2 | 38138728 | 38141609 | 3 | C0PG05_MAIZE | hypothetical protein LOC100383438 [Source:RefSeq peptide;Acc:NP_001169559] |
| 2 | 3188143 | 3188640 | 3 | Q9SBI5_MAIZE | invertase cell wall4 (incw4), mRNA [Source:RefSeq DNA;Acc:NM_001111429] |
| 2 | 235064648 | 235066817 | 3 | C0PD40_MAIZE | hypothetical protein LOC100383110 [Source:RefSeq peptide;Acc:NP_001169249] |
| 2 | 234575111 | 234576854 | 3 | GRMZM2G408158 | No description |
| 2 | 234110805 | 234111845 | 3 | GRMZM5G817777 | No description |
| 2 | 232648880 | 232651058 | 3 | B6U7W3_MAIZE | nitrate and chloride transporter [Source:RefSeq peptide;Acc:NP_001152028] |
| 2 | 218217623 | 218219691 | 3 | C0PDM3_MAIZE | hypothetical protein LOC100383151 [Source:RefSeq peptide;Acc:NP_001169287] |
| 2 | 193842347 | 193843862 | 3 | B6TGF3_MAIZE | hypothetical protein LOC100276681 [Source:RefSeq peptide;Acc:NP_001143880] |
| 2 | 190503989 | 190505254 | 3 | B6U9W1_MAIZE | hypothetical protein LOC100278507 [Source:RefSeq peptide;Acc:NP_001145235] |
| 2 | 186311253 | 186312327 | 3 | GRMZM2G138396 | No description |
| 2 | 161198046 | 161199034 | 3 | B6T4R2_MAIZE | hypothetical protein LOC100279567 [Source:RefSeq peptide;Acc:NP_001146036] |
| 2 | 15993945 | 15998186 | 3 | GRMZM2G114276 | No description |
| 2 | 157236886 | 157238655 | 3 | C0P5N4_MAIZE | hypothetical protein LOC100382087 [Source:RefSeq peptide;Acc:NP_001168321] |
| 2 | 15243138 | 15244211 | 3 | GRMZM2G038835 | No description |
| 2 | 144800491 | 144803445 | 3 | C4J4N1_MAIZE | Putative uncharacterized protein [Source:UniProtKB/TrEMBL;Acc:C4J4N1] |
| 2 | 144719579 | 144721645 | 3 | GRMZM2G046201 | No description |
| 2 | 139643865 | 139645573 | 3 | GRMZM2G066213 | No description |
| 2 | 13615727 | 13617630 | 3 | B7ZWZ1_MAIZE | Putative uncharacterized protein [Source:UniProtKB/TrEMBL;Acc:B7ZWZ1] |
| 2 | 120436484 | 120438137 | 3 | AC235540.1_FG002 | No description |
| 3 | 53275060 | 53275241 | 27 | GRMZM2G453642 | No description |
| 3 | 3830165 | 3832582 | 4 | C0HFL4_MAIZE | hypothetical protein LOC100304424 [Source:RefSeq peptide;Acc:NP_001159330] |
| 3 | 229585101 | 229587515 | 4 | GRMZM2G166745 | No description |
| 3 | 203893426 | 203895144 | 4 | C0PE40_MAIZE | hypothetical protein LOC100383200 [Source:RefSeq peptide;Acc:NP_001169333] |
| 3 | 133782226 | 133785036 | 4 | C0PF49_MAIZE | hypothetical protein LOC100383326 [Source:RefSeq peptide;Acc:NP_001169455] |
| 3 | 7965296 | 7966901 | 3 | GRMZM2G367898 | No description |
| 3 | 4843609 | 4844855 | 3 | B6TNA8_MAIZE | meiosis 5 [Source:RefSeq peptide;Acc:NP_001150305] |
| 3 | 45046974 | 45048075 | 3 | B8A3Q0_MAIZE | hypothetical protein LOC100280416 [Source:RefSeq peptide;Acc:NP_001146811] |
| 3 | 32124585 | 32127541 | 3 | C0PLZ2_MAIZE | hypothetical protein LOC100384152 [Source:RefSeq peptide;Acc:NP_001170202] |
| 3 | 228373735 | 228375510 | 3 | B4FQV8_MAIZE | hypothetical protein LOC100272813 [Source:RefSeq peptide;Acc:NP_001140738] |
| 3 | 224401252 | 224402309 | 3 | GRMZM2G090725 | No description |
| 3 | 20704046 | 20705189 | 3 | B6SRF1_MAIZE | calmodulin-related protein 2, touch-induced [Source:RefSeq peptide;Acc:NP_001147546] |
| 3 | 205000836 | 205001393 | 3 | GRMZM2G035276 | No description |
| 3 | 201093504 | 201094651 | 3 | C0PGB3_MAIZE | hypothetical protein LOC100383482 [Source:RefSeq peptide;Acc:NP_001169601] |
| 3 | 199276253 | 199279073 | 3 | GRMZM2G177883 | No description |
| 3 | 197282787 | 197283632 | 3 | B4FGD6_MAIZE | hypothetical protein LOC100193809 [Source:RefSeq peptide;Acc:NP_001132364] |
| 3 | 195963141 | 195965558 | 3 | B6TR04_MAIZE | hypothetical protein LOC100277220 [Source:RefSeq peptide;Acc:NP_001144325] |
| 3 | 186563277 | 186563951 | 3 | GRMZM2G117022 | No description |
| 3 | 179218925 | 179220103 | 3 | B6SN54_MAIZE | hypothetical protein LOC100193545 [Source:RefSeq peptide;Acc:NP_001132128] |
| 3 | 172371359 | 172371478 | 3 | C0P5Q1_MAIZE | hypothetical protein LOC100382244 [Source:RefSeq peptide;Acc:NP_001168468] |
| 3 | 146250146 | 146253250 | 3 | GRMZM2G309512 | No description |
| 3 | 115005663 | 115007783 | 3 | GRMZM2G457411 | No description |
| 4 | 156341992 | 156342051 | 51 | AC216872.3_FG002 | No description |
| 4 | 38570272 | 38571827 | 5 | GRMZM2G123257 | No description |
| 4 | 48236235 | 48237904 | 4 | GRMZM2G007533 | No description |
| 4 | 41224384 | 41225559 | 4 | AC183521.2_FG002 | No description |
| 4 | 197054729 | 197054959 | 4 | C0HI85_MAIZE | hypothetical protein LOC100381708 [Source:RefSeq peptide;Acc:NP_001167988] |
| 4 | 181870770 | 181873108 | 4 | C0PHS9_MAIZE | Putative uncharacterized protein [Source:UniProtKB/TrEMBL;Acc:C0PHS9] |
| 4 | 97691197 | 97692512 | 3 | GRMZM2G047083 | No description |
| 4 | 77360900 | 77363311 | 3 | GRMZM2G103186 | No description |
| 4 | 62793116 | 62796058 | 3 | C0P632_MAIZE | Putative uncharacterized protein [Source:UniProtKB/TrEMBL;Acc:C0P632] |
| 4 | 38758715 | 38760499 | 3 | C4J2F4_MAIZE | MADS-box transcription factor 26 [Source:RefSeq peptide;Acc:NP_001148873] |
| 4 | 30678789 | 30680180 | 3 | GRMZM2G337387 | No description |
| 4 | 26653585 | 26655098 | 3 | B6U9N7_MAIZE | F-box domain containing protein [Source:RefSeq peptide;Acc:NP_001152150] |
| 4 | 238223028 | 238223657 | 3 | GRMZM2G042664 | No description |
| 4 | 237311806 | 237314176 | 3 | B6T208_MAIZE | 60S ribosomal protein L19-3 [Source:RefSeq peptide;Acc:NP_001150484] |
| 4 | 235118646 | 235119794 | 3 | GRMZM2G036711 | WRKY71 - superfamily of TFs having WRKY and zinc finger domains mRNA [Source:RefSeq DNA;Acc:NM_001154260] |
| 4 | 226067153 | 226068764 | 3 | B6SJB5_MAIZE | DNA binding protein [Source:RefSeq peptide;Acc:NP_001146992] |
| 4 | 199797647 | 199798802 | 3 | B8A391_MAIZE | hypothetical protein LOC100280335 [Source:RefSeq peptide;Acc:NP_001146733] |
| 4 | 190829435 | 190831394 | 3 | GRMZM2G159854 | No description |
| 4 | 175277250 | 175279264 | 3 | GRMZM2G301647 | No description |
| 4 | 174042012 | 174044883 | 3 | B6TJT9_MAIZE | hypothetical protein LOC100276859 [Source:RefSeq peptide;Acc:NP_001144035] |
| 4 | 168737104 | 168738634 | 3 | C3UZ62_MAIZE | CDPK protein [Source:RefSeq peptide;Acc:NP_001170479] |
| 4 | 158195520 | 158197471 | 3 | GRMZM2G156472 | No description |
| 4 | 145872778 | 145873161 | 3 | B7ZYQ2_MAIZE | hypothetical protein LOC100272578 [Source:RefSeq peptide;Acc:NP_001140515] |
| 4 | 145731702 | 145732412 | 3 | B4FQZ5_MAIZE | hypothetical protein LOC100272825 (LOC100272825), mRNA [Source:RefSeq DNA;Acc:NM_001147278] |
| 4 | 13946719 | 13948647 | 3 | C0P793_MAIZE | hypothetical protein LOC100279575 (LOC100279575), mRNA [Source:RefSeq DNA;Acc:NM_001152572] |
| 4 | 131700745 | 131701313 | 3 | GRMZM2G060023 | No description |
| 4 | 111895429 | 111896985 | 3 | GRMZM2G127690 | No description |
| 5 | 54214582 | 54214909 | 39 | GRMZM2G022881 | No description |
| 5 | 118615364 | 118615685 | 38 | GRMZM2G033658 | No description |
| 5 | 53276767 | 53277121 | 34 | GRMZM2G047160 | No description |
| 5 | 212027167 | 212030047 | 5 | B7ZYS5_MAIZE | hypothetical protein LOC100279566 [Source:RefSeq peptide;Acc:NP_001146035] |
| 5 | 24609083 | 24610156 | 4 | B4FYA7_MAIZE | hypothetical protein LOC100273954 [Source:RefSeq peptide;Acc:NP_001141815] |
| 5 | 136581652 | 136583153 | 4 | GRMZM2G147172 | No description |
| 5 | 86071110 | 86071928 | 3 | B4F7T8_MAIZE | hypothetical protein LOC100191152 [Source:RefSeq peptide;Acc:NP_001130060] |
| 5 | 70772846 | 70776203 | 3 | B6U6I8_MAIZE | ethanolaminephosphotransferase [Source:RefSeq peptide;Acc:NP_001151915] |
| 5 | 69114970 | 69116904 | 3 | B6SRR0_MAIZE | glycerol-3-phosphate acyltransferase 8 [Source:RefSeq peptide;Acc:NP_001147442] |
| 5 | 65222254 | 65222505 | 3 | B4FNE5_MAIZE | hypothetical protein LOC100272396 [Source:RefSeq peptide;Acc:NP_001140348] |
| 5 | 5888925 | 5890767 | 3 | B4F8Q3_MAIZE | hypothetical protein LOC100191409 [Source:RefSeq peptide;Acc:NP_001130315] |
| 5 | 56911934 | 56912728 | 3 | GRMZM2G329999 | No description |
| 5 | 54171713 | 54173914 | 3 | B4F9C6_MAIZE | hypothetical protein LOC100191584 [Source:RefSeq peptide;Acc:NP_001130486] |
| 5 | 38938006 | 38938735 | 3 | GRMZM2G003274 | No description |
| 5 | 33641516 | 33644808 | 3 | GRMZM2G144420 | No description |
| 5 | 27060705 | 27062618 | 3 | GRMZM2G117963 | No description |
| 5 | 215300432 | 215302809 | 3 | C4J3R9_MAIZE | Putative uncharacterized protein [Source:UniProtKB/TrEMBL;Acc:C4J3R9] |
| 5 | 214535623 | 214537417 | 3 | GRMZM2G163195 | No description |
| 5 | 20986643 | 20987466 | 3 | C0PMR7_MAIZE | Putative uncharacterized protein [Source:UniProtKB/TrEMBL;Acc:C0PMR7] |
| 5 | 206634340 | 206635150 | 3 | GRMZM2G127632 | No description |
| 5 | 201866687 | 201867196 | 3 | AC216731.3_FG001 | No description |
| 5 | 196249840 | 196251589 | 3 | B4FVC0_MAIZE | plant-specific domain TIGR01568 family protein [Source:RefSeq peptide;Acc:NP_001148277] |
| 5 | 19524083 | 19526957 | 3 | GRMZM2G178753 | No description |
| 5 | 192404507 | 192405487 | 3 | GRMZM2G430392 | No description |
| 5 | 188472401 | 188474104 | 3 | C4J4N5_MAIZE | Putative uncharacterized protein [Source:UniProtKB/TrEMBL;Acc:C4J4N5] |
| 5 | 187848233 | 187849324 | 3 | GRMZM2G159918 | No description |
| 5 | 178351695 | 178352966 | 3 | B4FAI6_MAIZE | hypothetical protein LOC100191881 [Source:RefSeq peptide;Acc:NP_001130777] |
| 5 | 176991826 | 176993860 | 3 | B6TVL1_MAIZE | hypothetical protein LOC100279555 (LOC100279555), mRNA [Source:RefSeq DNA;Acc:NM_001152552] |
| 5 | 168532212 | 168534340 | 3 | Q2P9N4_MAIZE | putative glycosyltransferase [Source:RefSeq peptide;Acc:NP_001105849] |
| 5 | 15770557 | 15774111 | 3 | C4JAK4_MAIZE | Putative uncharacterized protein [Source:UniProtKB/TrEMBL;Acc:C4JAK4] |
| 5 | 153945630 | 153948183 | 3 | GRMZM2G119623 | No description |
| 5 | 151884598 | 151887217 | 3 | GRMZM2G176206 | No description |
| 5 | 140893993 | 140895248 | 3 | GRMZM2G000071 | No description |
| 5 | 112753088 | 112754513 | 3 | B6TP81_MAIZE | ring canal kelch [Source:RefSeq peptide;Acc:NP_001150390] |
| 6 | 98571470 | 98574524 | 6 | GRMZM2G129642 | hypothetical protein LOC100279621 (LOC100279621), mRNA [Source:RefSeq DNA;Acc:NM_001152617] |
| 6 | 140854200 | 140855687 | 6 | GRMZM2G313359 | No description |
| 6 | 95940460 | 95946990 | 4 | GRMZM2G171328 | No description |
| 6 | 153784123 | 153787056 | 4 | GRMZM2G125034 | No description |
| 6 | 88745204 | 88746905 | 3 | B6T3B2_MAIZE | hypothetical protein LOC100272890 [Source:RefSeq peptide;Acc:NP_001140815] |
| 6 | 73908832 | 73911103 | 3 | B4FA12_MAIZE | hypothetical protein LOC100191750 [Source:RefSeq peptide;Acc:NP_001130649] |
| 6 | 62544841 | 62545515 | 3 | B4FCB9_MAIZE | hypothetical protein LOC100192700 [Source:RefSeq peptide;Acc:NP_001131375] |
| 6 | 61601766 | 61602989 | 3 | C4J5P0_MAIZE | hypothetical protein LOC100272818 [Source:RefSeq peptide;Acc:NP_001140743] |
| 6 | 45710876 | 45712069 | 3 | GRMZM2G458548 | No description |
| 6 | 2589787 | 2592612 | 3 | GRMZM2G116685 | hypothetical protein LOC100384671 (LOC100384671), mRNA [Source:RefSeq DNA;Acc:NM_001177154] |
| 6 | 20413491 | 20414357 | 3 | GRMZM2G007944 | No description |
| 6 | 165800173 | 165802545 | 3 | GRMZM2G156620 | No description |
| 6 | 165625185 | 165626620 | 3 | GRMZM2G456175 | No description |
| 6 | 158476701 | 158477797 | 3 | GRMZM2G134846 | No description |
| 6 | 150074501 | 150076557 | 3 | GRMZM2G129288 | hypothetical protein LOC100383825 (LOC100383825), mRNA [Source:RefSeq DNA;Acc:NM_001176457] |
| 6 | 147690396 | 147690950 | 3 | B6SUN1_MAIZE | anther-specific proline-rich protein APG [Source:RefSeq peptide;Acc:NP_001147753] |
| 6 | 14678967 | 14680192 | 3 | B4FK30_MAIZE | hypothetical protein LOC100216863 [Source:RefSeq peptide;Acc:NP_001136726] |
| 6 | 142641698 | 142644047 | 3 | GRMZM2G431861 | No description |
| 6 | 138284904 | 138286392 | 3 | GRMZM2G447480 | No description |
| 6 | 129070873 | 129071673 | 3 | GRMZM2G447791 | No description |
| 6 | 128299646 | 128301084 | 3 | GRMZM2G044442 | No description |
| 6 | 124803945 | 124805261 | 3 | C0P928_MAIZE | fasciclin-like arabinogalactan protein 8 [Source:RefSeq peptide;Acc:NP_001147121] |
| 6 | 124299277 | 124300947 | 3 | GRMZM2G402538 | No description |
| 6 | 123777182 | 123778357 | 3 | Q6TM44_MAIZE | hypothetical protein LOC100272933 [Source:RefSeq peptide;Acc:NP_001140857] |
| 6 | 122958232 | 122958957 | 3 | GRMZM5G850036 | No description |
| 6 | 119395032 | 119397840 | 3 | C0P6K2_MAIZE | Putative uncharacterized protein [Source:UniProtKB/TrEMBL;Acc:C0P6K2] |
| 6 | 114571949 | 114573320 | 3 | B6UBT3_MAIZE | TMV response-related protein [Source:RefSeq peptide;Acc:NP_001152287] |
| 6 | 112434261 | 112435857 | 3 | GRMZM2G383680 | No description |
| 6 | 105537997 | 105540201 | 3 | B4F816_MAIZE | hypothetical protein LOC100191218 [Source:RefSeq peptide;Acc:NP_001130124] |
| 7 | 53726306 | 53726502 | 60 | GRMZM2G000011 | No description |
| 7 | 7086499 | 7086811 | 17 | GRMZM2G102668 | No description |
| 7 | 156120965 | 156122531 | 4 | GRMZM2G465987 | No description |
| 7 | 154625007 | 154625967 | 4 | GRMZM2G309258 | No description |
| 7 | 150038890 | 150042852 | 4 | C0P3Z6_MAIZE | hypothetical protein LOC100382055 [Source:RefSeq peptide;Acc:NP_001168291] |
| 7 | 125118573 | 125121392 | 4 | GRMZM2G009166 | No description |
| 7 | 9794502 | 9796890 | 3 | GRMZM2G406746 | No description |
| 7 | 9197711 | 9198396 | 3 | C0P3F4_MAIZE | hypothetical protein LOC100381993 [Source:RefSeq peptide;Acc:NP_001168233] |
| 7 | 82941333 | 82943672 | 3 | GRMZM2G115773 | No description |
| 7 | 804934 | 806800 | 3 | C0PJ78_MAIZE | hypothetical protein LOC100383810 [Source:RefSeq peptide;Acc:NP_001169913] |
| 7 | 79095632 | 79099586 | 3 | GRMZM2G092604 | No description |
| 7 | 5328420 | 5329301 | 3 | GRMZM2G442791 | No description |
| 7 | 45384708 | 45386649 | 3 | GRMZM2G354772 | No description |
| 7 | 31519120 | 31521422 | 3 | C4J4G8_MAIZE | Putative uncharacterized protein [Source:UniProtKB/TrEMBL;Acc:C4J4G8] |
| 7 | 30875070 | 30875597 | 3 | GRMZM5G817964 | No description |
| 7 | 26487882 | 26488851 | 3 | Q9LLI2_MAIZE | cellulose synthase8 [Source:RefSeq peptide;Acc:NP_001104958] |
| 7 | 170948243 | 170948778 | 3 | GRMZM2G036872 | No description |
| 7 | 170395573 | 170397112 | 3 | GRMZM2G019666 | No description |
| 7 | 167341222 | 167344151 | 3 | GRMZM2G459820 | No description |
| 7 | 165744759 | 165746434 | 3 | Q5GAU2_MAIZE | F-box protein [Source:UniProtKB/TrEMBL;Acc:Q5GAU2] |
| 7 | 164773048 | 164774636 | 3 | GRMZM2G049177 | No description |
| 7 | 158368564 | 158370335 | 3 | B6SU23_MAIZE | Nodulation signaling pathway 2 protein [Source:UniProtKB/TrEMBL;Acc:B6SU23] |
| 7 | 137824231 | 137825672 | 3 | B6T7R4_MAIZE | MTD1 [Source:RefSeq peptide;Acc:NP_001148854] |
| 7 | 137548199 | 137549102 | 3 | GRMZM2G423567 | No description |
| 7 | 134038900 | 134039768 | 3 | B6SUL7_MAIZE | TMV response-related protein [Source:RefSeq peptide;Acc:NP_001147747] |
| 7 | 117847534 | 117848780 | 3 | B4FR94_MAIZE | hypothetical protein LOC100279914 [Source:RefSeq peptide;Acc:NP_001146338] |
| 7 | 115309658 | 115310466 | 3 | B1Q040_MAIZE | WRKY DNA-binding protein [Source:RefSeq peptide;Acc:NP_001120723] |
| 7 | 103876337 | 103878886 | 3 | B6SWJ0_MAIZE | ubiquitin-protein ligase [Source:RefSeq peptide;Acc:NP_001147953] |
| 8 | 173719258 | 173720379 | 9 | GRMZM2G082490 | No description |
| 8 | 166827262 | 166828995 | 5 | GRMZM2G026847 | No description |
| 8 | 140005748 | 140009278 | 4 | GRMZM5G852378 | No description |
| 8 | 120777110 | 120778906 | 4 | B6U746_MAIZE | metacaspase type II [Source:RefSeq peptide;Acc:NP_001151968] |
| 8 | 78856418 | 78857806 | 3 | C0PEB2_MAIZE | hypothetical protein LOC100383225 [Source:RefSeq peptide;Acc:NP_001169358] |
| 8 | 65981741 | 65983349 | 3 | B6UFB5_MAIZE | indole-3-acetate beta-glucosyltransferase [Source:RefSeq peptide;Acc:NP_001152529] |
| 8 | 4258017 | 4259694 | 3 | GRMZM2G012728 | No description |
| 8 | 27215345 | 27216282 | 3 | B4FTG2_MAIZE | 3-methyl-2-oxobutanoate hydroxymethyltransferase [Source:RefSeq peptide;Acc:NP_001150103] |
| 8 | 23799793 | 23801891 | 3 | GRMZM5G861077 | No description |
| 8 | 20081634 | 20083207 | 3 | GRMZM2G096904 | No description |
| 8 | 18442050 | 18444157 | 3 | C0P7G2_MAIZE | hypothetical protein LOC100382417 [Source:RefSeq peptide;Acc:NP_001168631] |
| 8 | 171149687 | 171150978 | 3 | B6THT9_MAIZE | hypothetical protein LOC100276764 [Source:RefSeq peptide;Acc:NP_001143951] |
| 8 | 169789008 | 169790495 | 3 | B6SVH9_MAIZE | hypothetical protein LOC100275561 [Source:RefSeq peptide;Acc:NP_001143087] |
| 8 | 166836625 | 166838331 | 3 | B6U5U9_MAIZE | sialyltransferase-like protein [Source:RefSeq peptide;Acc:NP_001151877] |
| 8 | 16352457 | 16353551 | 3 | C0HHG7_MAIZE | hypothetical protein LOC100381619 [Source:RefSeq peptide;Acc:NP_001167907] |
| 8 | 159390018 | 159392771 | 3 | GRMZM2G303587 | No description |
| 8 | 157462876 | 157464783 | 3 | GRMZM2G059117 | No description |
| 8 | 157295026 | 157296818 | 3 | GRMZM2G118619 | No description |
| 8 | 153015039 | 153016559 | 3 | GRMZM2G173965 | No description |
| 8 | 149148002 | 149149318 | 3 | GRMZM2G075058 | No description |
| 8 | 142476468 | 142478055 | 3 | C0HG35_MAIZE | hypothetical protein LOC100381450 [Source:RefSeq peptide;Acc:NP_001167759] |
| 8 | 137281378 | 137281854 | 3 | GRMZM2G115059 | No description |
| 8 | 12609776 | 12611068 | 3 | GRMZM2G133552 | No description |
| 8 | 121435529 | 121437647 | 3 | B6U0X3_MAIZE | hypothetical protein LOC100191999 [Source:RefSeq peptide;Acc:NP_001130895] |
| 9 | 131256514 | 131258669 | 12 | GRMZM2G042510 | No description |
| 9 | 23008651 | 23010843 | 5 | B4FBK7_MAIZE | hypothetical protein LOC100192500 [Source:RefSeq peptide;Acc:NP_001131192] |
| 9 | 87811933 | 87813386 | 4 | GRMZM2G475884 | No description |
| 9 | 2258593 | 2259952 | 4 | B4FZU1_MAIZE | hypothetical protein LOC100274259 [Source:RefSeq peptide;Acc:NP_001142095] |
| 9 | 14113094 | 14115206 | 4 | GRMZM2G009139 | No description |
| 9 | 138763994 | 138765008 | 4 | GRMZM2G162451 | No description |
| 9 | 121937248 | 121938756 | 4 | B6SWD3_MAIZE | glucan endo-1,3-beta-glucosidase 5 [Source:RefSeq peptide;Acc:NP_001147933] |
| 9 | 112004919 | 112007822 | 4 | GRMZM2G049229 | No description |
| 9 | 106624342 | 106628815 | 4 | C0PE38_MAIZE | Putative uncharacterized protein [Source:UniProtKB/TrEMBL;Acc:C0PE38] |
| 9 | 7614057 | 7616811 | 3 | B6TIA5_MAIZE | LOC100283424 [Source:RefSeq peptide;Acc:NP_001149797] |
| 9 | 26478611 | 26479384 | 3 | GRMZM2G393146 | No description |
| 9 | 20337422 | 20338112 | 3 | B6SYY9_MAIZE | sulfate transporter 3.4 [Source:RefSeq peptide;Acc:NP_001148179] |
| 9 | 154307169 | 154308189 | 3 | GRMZM5G829967 | No description |
| 9 | 146890292 | 146891017 | 3 | GRMZM2G423331 | No description |
| 9 | 146226888 | 146229140 | 3 | C4J6V0_MAIZE | Putative uncharacterized protein [Source:UniProtKB/TrEMBL;Acc:C4J6V0] |
| 9 | 134917247 | 134918998 | 3 | GRMZM2G473356 | No description |
| 9 | 124946862 | 124947851 | 3 | GRMZM2G438121 | No description |
| 9 | 120747555 | 120750044 | 3 | GRMZM2G154029 | hypothetical protein LOC100382122 (LOC100382122), mRNA [Source:RefSeq DNA;Acc:NM_001174883] |
| 9 | 11777826 | 11780181 | 3 | Q8W1D3_MAIZE | Serine threonine kinase [Source:UniProtKB/TrEMBL;Acc:Q8W1D3] |
| 9 | 101690078 | 101690780 | 3 | B6TUQ3_MAIZE | Putative uncharacterized protein [Source:UniProtKB/TrEMBL;Acc:B6TUQ3] |
| 9 | 100188448 | 100191000 | 3 | GRMZM2G334592 | No description |
| 10 | 49787631 | 49787810 | 13 | GRMZM2G119008 | No description |
| 10 | 113152065 | 113153572 | 6 | GRMZM2G006490 | No description |
| 10 | 2654215 | 2657791 | 4 | GRMZM2G127393 | No description |
| 10 | 70919582 | 70921917 | 3 | C0PHP0_MAIZE | hypothetical protein LOC100383614 [Source:RefSeq peptide;Acc:NP_001169733] |
| 10 | 68924719 | 68926477 | 3 | B4FTH5_MAIZE | LOC100285134 [Source:RefSeq peptide;Acc:NP_001151500] |
| 10 | 146335484 | 146336399 | 3 | GRMZM2G400109 | No description |
| 10 | 141564224 | 141564697 | 3 | B4FMV5_MAIZE | hypothetical protein LOC100272301 [Source:RefSeq peptide;Acc:NP_001140259] |
| 10 | 137458157 | 137459014 | 3 | AC209206.3_FG014 | No description |
| 10 | 133302514 | 133303662 | 3 | GRMZM2G001035 | No description |
| 10 | 125538211 | 125541575 | 3 | C0P351_MAIZE | hypothetical protein LOC100193538 [Source:RefSeq peptide;Acc:NP_001132121] |
| 10 | 12166679 | 12168399 | 3 | GRMZM2G094375 | No description |
| 10 | 120671025 | 120672700 | 3 | GRMZM2G079490 | No description |
| 10 | 117570464 | 117571861 | 3 | GRMZM2G072322 | No description |
| 10 | 114289641 | 114289799 | 3 | Q9ZTQ5_MAIZE | invertase cell wall3 [Source:RefSeq peptide;Acc:NP_001104898] |
| 10 | 114237661 | 114239362 | 3 | B6U1H5_MAIZE | beta-fructofuranosidase, insoluble isoenzyme 2 [Source:RefSeq peptide;Acc:NP_001151535] |
| 10 | 112937715 | 112938912 | 3 | GRMZM2G368047 | No description |
| 10 | 106434010 | 106435286 | 3 | B4FJT5_MAIZE | hypothetical protein LOC100216803 [Source:RefSeq peptide;Acc:NP_001136674] |
