# Supplementary material for: Deep Sequencing of RNA from Ancient Maize Kernels
Source: PLoS One. 2013 Jan 11;8(1):e50961. doi: 10.1371/journal.pone.0050961 (PMC3543400; doi:10.1371/journal.pone.0050961)
Supplement: Table S6 — Functionally annotated exon hits for Arizonan kernel 935230. (DOCX) [file pone.0050961.s012.docx]

**Table S6**

| Chr | Start | End | Reads | ID | Description |
| --- | --- | --- | --- | --- | --- |
| 1 | 51463393 | 51463721 | 46 | GRMZM2G177990 | No description |
| 1 | 168908705 | 168909014 | 44 | GRMZM2G052361 | No description |
| 1 | 161046218 | 161046528 | 20 | GRMZM2G130258 | No description |
| 1 | 166323758 | 166324039 | 20 | GRMZM2G427402 | No description |
| 1 | 176412407 | 176412500 | 8 | GRMZM2G095389 | No description |
| 1 | 166323451 | 166323614 | 7 | GRMZM2G427402 | No description |
| 1 | 4052734 | 4055543 | 4 | B4FZU2_MAIZE | hypothetical protein LOC100274260 [Source:RefSeq peptide;Acc:NP_001142096] |
| 1 | 1779988 | 1782907 | 3 | GRMZM2G161540 | hypothetical protein LOC100381635 (LOC100381635), mRNA [Source:RefSeq DNA;Acc:NM_001174451] |
| 1 | 33955492 | 33956487 | 3 | GRMZM2G321033 | No description |
| 1 | 188457694 | 188458952 | 3 | GRMZM2G022499 | No description |
| 1 | 262456286 | 262459646 | 3 | GRMZM2G351304 | No description |
| 2 | 48392981 | 48393259 | 27 | GRMZM2G112649 | No description |
| 2 | 14313150 | 14313434 | 24 | GRMZM2G070188 | No description |
| 2 | 41484111 | 41485573 | 3 | C0PBQ2_MAIZE | hypothetical protein LOC100382921 [Source:RefSeq peptide;Acc:NP_001169080] |
| 2 | 196137056 | 196139123 | 3 | GRMZM2G133310 | No description |
| 2 | 212531770 | 212532876 | 3 | B4FA05_MAIZE | hypothetical protein LOC100191746 [Source:RefSeq peptide;Acc:NP_001130645] |
| 3 | 53275060 | 53275241 | 24 | GRMZM2G453642 | No description |
| 3 | 92261956 | 92264766 | 3 | GRMZM2G080503 | No description |
| 3 | 145729325 | 145730140 | 3 | GRMZM2G174274 | No description |
| 3 | 160575857 | 160576586 | 3 | B4FAB7_MAIZE | hypothetical protein LOC100191828 [Source:RefSeq peptide;Acc:NP_001130724] |
| 3 | 170538883 | 170540691 | 3 | GRMZM2G316163 | No description |
| 3 | 173801386 | 173803474 | 3 | B6SRU6_MAIZE | amidophosphoribosyltransferase [Source:RefSeq peptide;Acc:NP_001147451] |
| 3 | 178805073 | 178806637 | 3 | GRMZM5G832491 | No description |
| 3 | 222594453 | 222596230 | 3 | AC191045.3_FG006 | No description |
| 4 | 156341992 | 156342051 | 52 | AC216872.3_FG002 | No description |
| 4 | 159507898 | 159508226 | 6 | GRMZM2G005721 | No description |
| 4 | 38235715 | 38237197 | 3 | GRMZM2G112792 | No description |
| 4 | 62793116 | 62796058 | 3 | C0P632_MAIZE | Putative uncharacterized protein [Source:UniProtKB/TrEMBL;Acc:C0P632] |
| 4 | 123666105 | 123668045 | 3 | GRMZM2G000380 | No description |
| 4 | 155910424 | 155911806 | 3 | B6SMS1_MAIZE | RING zinc finger protein-like [Source:RefSeq peptide;Acc:NP_001147218] |
| 4 | 174777312 | 174780409 | 3 | B4F9K7_MAIZE | LOC732831 [Source:RefSeq peptide;Acc:NP_001105911] |
| 5 | 54214582 | 54214909 | 38 | GRMZM2G022881 | No description |
| 5 | 53276767 | 53277121 | 29 | GRMZM2G047160 | No description |
| 5 | 118615364 | 118615685 | 27 | GRMZM2G033658 | No description |
| 5 | 19810224 | 19810765 | 7 | GRMZM2G140269 | No description |
| 5 | 38938006 | 38938735 | 6 | GRMZM2G003274 | No description |
| 5 | 49824975 | 49825688 | 4 | GRMZM2G477829 | No description |
| 5 | 6315211 | 6316774 | 3 | GRMZM2G035103 | No description |
| 5 | 7594371 | 7595057 | 3 | B6TZF1_MAIZE | transmembrane BAX inhibitor motif-containing protein 4 [Source:RefSeq peptide;Acc:NP_001151352] |
| 5 | 33641516 | 33644808 | 3 | GRMZM2G144420 | No description |
| 5 | 180632305 | 180634311 | 3 | B6TPG0_MAIZE | elongation factor Tu [Source:RefSeq peptide;Acc:NP_001150410] |
| 5 | 193452399 | 193454440 | 3 | B4FJ20_MAIZE | hypothetical protein LOC100216652 [Source:RefSeq peptide;Acc:NP_001136535] |
| 5 | 199157014 | 199158382 | 3 | B4FK72_MAIZE | hypothetical protein LOC100216881 [Source:RefSeq peptide;Acc:NP_001136740] |
| 5 | 214535623 | 214537417 | 3 | GRMZM2G163195 | No description |
| 6 | 11754785 | 11755390 | 5 | GRMZM2G169834 | No description |
| 6 | 20413491 | 20414357 | 4 | GRMZM2G007944 | No description |
| 6 | 79553937 | 79555390 | 4 | GRMZM2G119865 | No description |
| 6 | 140854200 | 140855687 | 3 | GRMZM2G313359 | No description |
| 6 | 160013893 | 160015192 | 3 | B6SLI7_MAIZE | CCCH transcription factor [Source:RefSeq peptide;Acc:NP_001147375] |
| 6 | 160621048 | 160622526 | 3 | C0PDC7_MAIZE | heat-shock protein 101 [Source:RefSeq peptide;Acc:NP_001104935] |
| 6 | 164143733 | 164144243 | 3 | B6SW78_MAIZE | hypothetical protein LOC100274290 [Source:RefSeq peptide;Acc:NP_001142126] |
| 6 | 165162158 | 165164637 | 3 | C0P9H4_MAIZE | hypothetical protein LOC100382638 [Source:RefSeq peptide;Acc:NP_001168833] |
| 7 | 53726306 | 53726502 | 54 | GRMZM2G000011 | No description |
| 7 | 7086499 | 7086811 | 21 | GRMZM2G102668 | No description |
| 7 | 153248973 | 153249284 | 4 | GRMZM2G075826 | No description |
| 7 | 85262752 | 85264684 | 3 | GRMZM2G418153 | No description |
| 7 | 137701178 | 137701561 | 3 | AC225790.2_FG003 | No description |
| 8 | 43075218 | 43076172 | 3 | GRMZM2G035542 | No description |
| 8 | 72298289 | 72300367 | 3 | B6TTV8_MAIZE | hypothetical protein LOC100277394 [Source:RefSeq peptide;Acc:NP_001144434] |
| 9 | 131256514 | 131258669 | 6 | GRMZM2G042510 | No description |
| 9 | 20747300 | 20749031 | 3 | B4FVI7_MAIZE | hexose carrier protein HEX6 [Source:RefSeq peptide;Acc:NP_001148202] |
| 9 | 37538687 | 37540567 | 3 | B4G0H1_MAIZE | hypothetical protein LOC100274364 [Source:RefSeq peptide;Acc:NP_001142196] |
| 9 | 57011529 | 57013736 | 3 | Q6Y3I1_MAIZE | multidrug resistance associated protein 1 [Source:RefSeq peptide;Acc:NP_001105942] |
| 9 | 102941138 | 102942491 | 3 | GRMZM2G152120 | No description |
| 9 | 134769888 | 134770264 | 3 | C0PNH4_MAIZE | hypothetical protein LOC100277158 [Source:RefSeq peptide;Acc:NP_001144279] |
| 10 | 49787631 | 49787810 | 18 | GRMZM2G119008 | No description |
| 10 | 113152065 | 113153572 | 9 | GRMZM2G006490 | No description |
| 10 | 4098565 | 4099212 | 3 | GRMZM2G467576 | No description |
