# Supplementary material for: Deep Sequencing of RNA from Ancient Maize Kernels
Source: PLoS One. 2013 Jan 11;8(1):e50961. doi: 10.1371/journal.pone.0050961 (PMC3543400; doi:10.1371/journal.pone.0050961)
Supplement: Table S7 — Repeat content profiles of HiSeq cDNA and GAIIx DNA sequences using RepeatMasker. (DOCX) [file pone.0050961.s013.docx]

**Table S7**

|  | **Number of elements** | | | **Length occupied (bp)** | | | **Percentage of sequence (%)** | | |
| --- | --- | --- | --- | --- | --- | --- | --- | --- | --- |
|  | **AZ shotgun** | **935130** | **935230** | **AZ shotgun** | **935130** | **935230** | **AZ shotgun** | **935130** | **935230** |
| **Retroelements** | **45,689** | **57** | **886** | **1,941,117** | **37,987** | **2,637** | **47.88** | **0.05** | **0.01** |
| SINEs | 8 | - | - | 333 | - | - | 0.01 | - | - |
| LINEs | 277 | - | - | 11,691 | - | - | 0.29 | - | - |
| RTE/Bov-B | 51 | - | - | 2,160 | - | - | 0.05 | - | - |
| L1/CIN4 | 226 | - | - | 9,531 | - | - | 0.24 | - | - |
| LTR elements | 45,404 | 57 | 886 | 1,929,093 | 37,987 | 2,637 | 47.58 | 0.05 | 0.01 |
| Ty1/Copia | 13,890 | 9 | 82 | 603,475 | 3,604 | 327 | 14.88 | - | - |
| Gypsy/DIRS1 | 31,460 | 41 | 679 | 1,323,428 | 27,419 | 1,943 | 32.64 | 0.04 | 0.01 |
| DNA transposons | 2,076 | - | 38 | 87,366 | 1,310 | - | 2.15 | - | - |
| hobo-Activator | 111 | - | 25 | 4,727 | 818 | - | 0.12 | - | - |
| Tc1-IS630-Pogo | 1 | - | - | 40 | - | - | - | - | - |
| En-Spm | 1,545 | - | - | 64,724 | - | - | 1.6 | - | - |
| MuDR-IS905 | 322 | - | 13 | 13,765 | 492 | - | 0.34 | - | - |
| Tourist/Harbinger | 90 | - | - | 3,855 | - | - | 0.1 | - | - |
| Unclassified | 224 | 8 | 2 | 9,391 | 182 | 710 | 0.23 | - | - |
| **Total interspersed repeats** |  |  |  | **2,037,874** | **39,479** | **3,347** | **50.26** | **0.05** | **0.01** |
| **Small RNA** | **224** | **459,802** | **988,699** | **9,710** | **64,585,935** | **25,848,184** | **0.24** | **89.04** | **72.42** |
| Satellites | 780 | 4 | 513 | 33,655 | 15,456 | 134 | 0.83 | 0.02 | - |
| Simple repeats | 119 | 2,846 | 1,646 | 4,205 | 59,847 | 97,387 | 0.1 | 0.08 | 0.27 |
| Low complexity | 27 | 186 | 155 | 907 | 3,734 | 4,682 | 0.02 | 0.01 | 0.01 |
